# Effects of soil and atmospheric drought on intra-annual δ^13^C patterns in tree rings

**DOI:** 10.1093/treephys/tpaf120

**Published:** 2025-09-27

**Authors:** Valentina Vitali, Jernej Jevšenak, Georg von Arx, Marina Fonti, Meisha Holloway-Phillips, Rubén D Manzanedo, Kerstin Treydte, Lorenz Walthert, Roman Zweifel, Matthias Saurer

**Affiliations:** Department of Environmental Systems Science, Forest Ecology, Institute of Terrestrial Ecosystems, ETH Zürich, Universitätstrasse 16, Zürich 8092, Switzerland; Swiss Federal Institute for Forest, Snow and Landscape Research WSL, Zürcherstrasse 111, Birmensdorf 8903, Switzerland; Department of Forest and Landscape Planning and Monitoring, Slovenian Forestry Institute, Večna pot 2, Ljubljana 1000, Slovenia; TUM School of Life Sciences, Technical University of Munich, Alte Akademie 8, Freising 80333, Germany; Swiss Federal Institute for Forest, Snow and Landscape Research WSL, Zürcherstrasse 111, Birmensdorf 8903, Switzerland; Oeschger Centre for Climate Change Research, University of Bern, Falkenplatz 16, Bern 3012, Switzerland; Swiss Federal Institute for Forest, Snow and Landscape Research WSL, Zürcherstrasse 111, Birmensdorf 8903, Switzerland; Swiss Federal Institute for Forest, Snow and Landscape Research WSL, Zürcherstrasse 111, Birmensdorf 8903, Switzerland; Oeschger Centre for Climate Change Research, University of Bern, Falkenplatz 16, Bern 3012, Switzerland; Institute of Plant Sciences, University of Bern, Altenbergrain 21, Bern 3015, Switzerland; Swiss Federal Institute for Forest, Snow and Landscape Research WSL, Zürcherstrasse 111, Birmensdorf 8903, Switzerland; Oeschger Centre for Climate Change Research, University of Bern, Falkenplatz 16, Bern 3012, Switzerland; Swiss Federal Institute for Forest, Snow and Landscape Research WSL, Zürcherstrasse 111, Birmensdorf 8903, Switzerland; Swiss Federal Institute for Forest, Snow and Landscape Research WSL, Zürcherstrasse 111, Birmensdorf 8903, Switzerland; Swiss Federal Institute for Forest, Snow and Landscape Research WSL, Zürcherstrasse 111, Birmensdorf 8903, Switzerland

**Keywords:** carbon isotopes, intra-annual growth, laser ablation, tree-ring isotopes, water availability

## Abstract

High-resolution carbon isotope ratio (δ^13^C) measurements of tree rings have the potential to provide seasonal environmental information. However, due to the complexity of the wood formation processes, the reliability of this method for intra-seasonal reconstruction of growing conditions remains unclear. We therefore investigated the intra-annual variation of δ^13^C in tree rings of three conifer species (*Pinus sylvestris* L., *Picea abies* (L.) H. Karst., *Abies alba* Mill.) across sites from the Swiss Alps to assess their response to seasonal variation of soil water potential (SWP) and vapour pressure deficit (VPD). Intra-annual δ^13^C values at a resolution of 10 points per year were assessed using laser-ablation isotope-ratio mass spectrometry. Seasonal δ^13^C patterns were analysed for synchronicity across trees and species, and their correlation with on-site environmental variables was used to determine the driving factors of δ^13^C, to reconstruct growing-season dynamics, and to estimate the timings of the growth dynamics and the allocation of carbon to xylem formation. The δ^13^C patterns showed high synchronicity between species, with characteristic maxima in wet and dry years occurring in the middle of the ring and at the end of the ring, respectively. Seasonal δ^13^C variations reliably reflected atmospheric dryness. Higher than normal soil dryness hindered the integration of further fresh assimilates into the xylem, thus allowing the identification of species- and site-specific threshold conditions that disrupt wood formation. The δ^13^C of Scots pine shows the strongest correlations with VPD and SWP, making it an excellent indicator of environmental variability. Silver fir appeared to integrate carbon into xylem structural material over a longer season than the other conifers, whilst Norway spruce shows more plastic, site-specific responses to environmental conditions. In conclusion, we identify how atmospheric and soil drought jointly impact tree growth and intra-annual δ^13^C patterns across conifer species, offering valuable insights for climate reconstructions and wider applications in forest dynamics.

## Introduction

For several decades, stable isotope ratios of tree rings have been successfully used as reliable indicators of environment–plant interactions (McCarroll and Loader 2004), for the investigation of spatial hydro-climatic patterns at the continental level (Vitali et al. 2021), and for centennial and millennial climate reconstruction (Churakova Sidorova et al. 2020, Büntgen et al. 2021, Treydte et al. 2024). However, most of these studies have an annual or even multi-annual temporal resolution. Due to methodological and interpretation limitations, far less research has been conducted to explore the intra-annual variation in the carbon isotope ratio (δ^13^C), or relating δ^13^C seasonal dynamics and interactions between phenology and the timing of cell development. These processes play a major role in carbon formation and its integration into wood structures (Cuny et al. 2014*a*, Castagneri et al. 2017). In conifers, large, thin-walled earlywood tracheids are formed at a high rate in early spring (Cuny et al. 2014*a*). Smaller, thick-walled latewood cells are built at lower rates but may live up to several months and integrate environmental information over a much longer period (Schollaen et al. 2014). Lignification processes and the use of carbon from storage may further blur the cells’ environmental information inherited from the carbon used to form them (Kagawa et al. 2006). Earlywood and latewood exhibit distinct anatomical and density characteristics, which significantly influence the intra-seasonal δ^13^C signature in tree rings. These variations can affect the interpretation of carbon isotope records as indicators of physiological processes and environmental conditions. Such anatomical differentiation modulates carbon assimilation and allocation, underscoring the importance of separately analysing EW and LW to improve the accuracy of climate and ecophysiological reconstructions (Vaganov et al. 2009, Belmecheri et al. 2018). However, with a high-resolution analysis, it is possible to study δ^13^C variations as a continuous variable, thus considering the overlapping integration of carbon across the ring. Nonetheless, understanding the information retained in intra-annual δ^13^C patterns remains an open challenge, with critical ecological insights waiting to be uncovered.

The variation in δ^13^C in tree rings provides an integrated record of the ratio of intercellular to atmospheric CO_2_ concentrations during the period when the carbon was fixed, reflecting the balance between net CO_2_ assimilation and stomatal conductance (Farquhar et al. 1989). Thus, δ^13^C can be related to canopy functioning and gas exchange, which are directly impacted by environmental factors, with their relative importance depending on the general climate region and site conditions (Gessler et al. 2014). Under optimal conditions, xylem production uses recent assimilates, as sufficient new assimilates are provided by photosynthesis and the phloem transport is fully functional (Gessler et al. 2009). As such, the δ^13^C of sugars utilized for wood production should integrate seasonal information of growth conditions (Helle and Schleser 2004, Gessler et al. 2014), with varying latency (Gessler et al. 2009). However, that might not always be the case. Earlywood growth in spring can also be supported by remobilized storage compounds, with this process being more apparent in deciduous trees (Monserud and Marshall 2001, Helle and Schleser 2004, Gričar et al. 2022), than in conifers (Martínez-Sancho et al. 2022, Rinne-Garmston et al. 2023). Similarly, radial growth in summer can also depend on remobilized carbon under extreme heat and drought conditions, thus resulting in a decoupling of the δ^13^C tree-ring signal from current leaf-level physiology (Hentschel et al. 2016). These results have raised concerns about the impacts of biochemical processes on the intra-annual δ^13^C patterns in tree rings. Building on these mechanistic principles, intra-annual δ^13^C analyses aim to disentangle finer-scale physiological and environmental signals across the growing season.

Intra-annual δ^13^C measurements allow for investigating the gradual incorporation of carbon assimilates in woody tissues and the associated environmental signals recorded in tree-ring isotopes (Pérez-de-Lis et al. 2022), either by integrating isotopic measurements with xylogenesis data (Tang et al. 2022), or by comparing intra-annual δ^13^C profiles with source water and carbohydrate pools (Michelot-Antalik et al. 2019). Various studies have made use of high-resolution information by measuring tree-ring isotope ratios through laser ablation (Skomarkova et al. 2006, Schollaen et al. 2014, Fonti et al. 2018, Rinne-Garmston et al. 2023) or by manually split tree-rings (Kagawa et al. 2006, Martínez-Sancho et al. 2022), but were often been limited to single sites and species. It remains unknown how consistent these intra-annual isotope signals are between species at a site and between sites, and thus, how reliably the information on environmental conditions can be retrieved. The progress in laser-ablation isotope ratio mass-spectrometry (LA-IRMS), improves the capacity for efficient and high-resolution (30 μm), detailed and accurate intra-annual data (Saurer et al. 2023), boosting the application potential of intra-annual high-resolution δ^13^C analysis.

**Figure 1:**
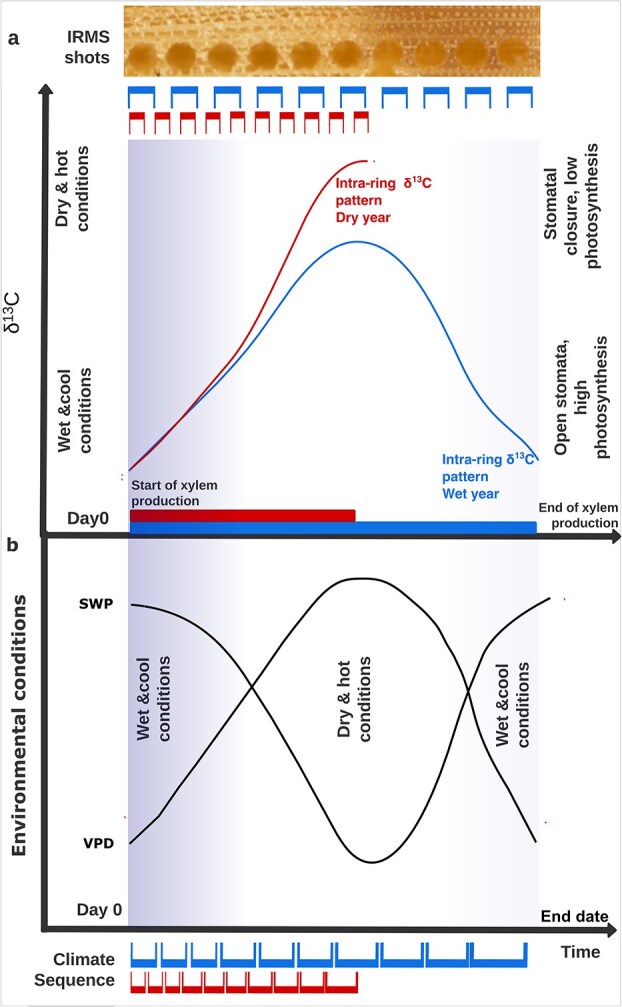
Conceptual diagram illustrating (a) δ^13^C intra-ring patterns observed from high-resolution LA-IRMS, with the 10-shots sequence creating a sequential sampling of the whole ring. The δ^13^C variation reflects seasonal changes in stomatal regulation and photosynthetic rates in response to (b) seasonal environmental conditions of atmospheric and soil dryness. In wet and cold years, high stomatal conductance and photosynthesis produce a characteristic ‘bell-shaped’ δ^13^C curve (blue δ^13^C line that spans from Day 0 to the end of the growing season). In contrast, hot and dry conditions induce tighter stomatal control and reduced photosynthesis, resulting in a steep δ^13^C increase from earlywood to latewood, forming a distinctive ‘monotonic rise’ pattern (red δ^13^C line, covering a shorter time span). (b) Schematic representation of the sequential division of seasonal environmental data into 10 windows, anchored to Day 0 (the onset of stem formation). The highest cross-correlation between δ^13^C and environmental variables, such as VPD and SWP, allows for the identification of the xylem-integrated seasonal environmental information. Red windows (smaller and with a shorter timespan) represent the selection for hot and dry years, corresponding to monotonic rise pattern of δ^13^C. This pattern indicates an earlier end of xylem formation compared to the blue windows (covering the whole theoretical timeframe), which represent wet and cool years, corresponding to ‘bell-shaped’ δ^13^C curves. This indicates longer xylem production, reflecting later-season environmental information.

Tree-ring δ^13^C variations are influenced by vapour pressure deficit (VPD), soil moisture (Tang et al. 2022) and drought conditions (Grossiord et al. 2014, Jucker et al. 2017), as plants respond to such conditions by closing their stomata to prevent water loss, especially in low soil water availability conditions, leading to higher δ^13^C values in tree rings (O’Leary 1995, Grossiord et al. 2014). The analysis of δ^13^C might, therefore, be a powerful tool for better understanding the combined effects of VPD and soil drought, which are known to affect many forests worldwide in recent decades (Novick et al. 2024). However, extremely dry conditions may not be recorded by δ^13^C if they coincide with growth cessation, i.e., stem growth must occur for the δ^13^C–environmental relationship to be recorded (Barbour and Song 2014). Conversely, adequate soil water availability reduces δ^13^C values by enhancing carbon assimilation (Gavrichkova et al. 2011). Therefore, we can explore seasonal growth kinetics and ^13^C isotopic composition by accounting for atmospheric and below-ground water availability (Zelenov et al. 2024). A seasonal shape has been shown in another LA-IRMS study, where a ‘bell-shaped’ pattern with low δ^13^C values (Figure 1, blue line) at the edges of the ring and maximum values in the middle was common (Tang et al. 2022). However, dry conditions should significantly modify the δ^13^C intra-annual pattern (Helle and Schleser 2004, Vaganov et al. 2009, Eglin et al. 2010, Etzold et al. 2022, Tang et al. 2022), interfering with the integration of the full seasonal trends, thus creating a particular ‘monotonic rise’ pattern (Figure 1, red line), where δ^13^C increases continuously from early to latewood (Xu et al. 2022). We hypothesize that these two δ^13^C shapes should be identifiable and shared for all species and sites with a clear distinction between wet and dry years (Figure 1). Thus, in generalizing this trends, the two δ^13^C shapes should be identifiable and shared for all species and sites with a clear distinction between wetter and drier than normal years (Figure 1). Under moist conditions the δ^13^C reflected seasonal trends, with lower (more negative) values in spring when conditions are moist and mild (leading to an ‘open stomata’ signal and thus ^13^C depletion), higher values during summer when it is dry and hot (leading to a ‘closed stomata’ signal, resulting in ^13^C enrichment) and lower values again in autumn when the environmental conditions return to moist and mild, resulting in a clear ‘bell-shaped’ pattern. In the case where δ^13^C increases continuously from early to latewood, it creates a particular ‘monotonic rise’ pattern. Thus, by identifying the different time integrations between the two curves, we can infer the timing of wood formation or early growth stops due to dry conditions.

Species-specific differences should be taken into account as they are associated with inherent genetic differences in water-use efficiency and responses to environmental factors (Dubbert et al. 2012, Babst et al. 2013, Brinkmann et al. 2019). Nonetheless, although functional strategies are species-specific, strong synchronicity of the maximum rates of cambial activity between co-existing conifer species has been observed (Cuny et al. 2012), implying that a large part of their wood formation occurs in an overlapping period. Thus, by aligning the start of growing activity with the beginning of stem growth identified by dendrometer measurements, we can further pinpoint which environmental conditions impact tree growth and ecophysiological mechanisms (Zweifel et al. 2021*b*, Pérez-de-Lis et al. 2022).

To explore the variation of intra-annual δ^13^C in tree rings and its capacity to record seasonal variability of atmospheric and soil aridity and to tease apart the confounding effect of species-specific responses to environmental conditions, we investigated co-occurring conifer species (Scots pine (*Pinus sylvestris* L.), silver fir (*Abies alba* Mill.) and Norway spruce (*Picea abies* (L.) H. Karst.), at forest sites varying in water availability. This study benefits from a unique setup in place for the past 10 years, with on-site continuous measurements of environmental variables, including temperature, air moisture and soil water potential (SWP), and tree growth monitoring with dendrometers. We pair these variables with high-temporal resolution δ^13^C measurements from 10 years of tree rings, totalling 4000 isotope measurements. Following the ecophysiological understanding of δ^13^C signals in tree rings, we expect that the dynamic relationship between air and soil moisture and tree physiological functioning is directly linked to the intra-annual variation of δ^13^C (see Site- and species-specific intra-annual δ^13^C patterns). This approach will provide a comprehensive understanding of intra-seasonal dynamics between environmental conditions, tree physiology and δ^13^C variation within tree rings, addressing the following hypotheses:

Hp.1 Common δ^13^C intra-annual patterns, shaped by environmental drivers, are consistently observed across different conifer species and sites.

Hp.2 The interplay between soil and atmospheric dryness explains the distinct δ^13^C variations within annual tree rings.

Hp. 3 The intra-annual environmental changes recorded by δ^13^C vary along the ring and provide a temporal link to xylem formation.

## Materials and methods

### Site and tree species selection

**Figure 2:**
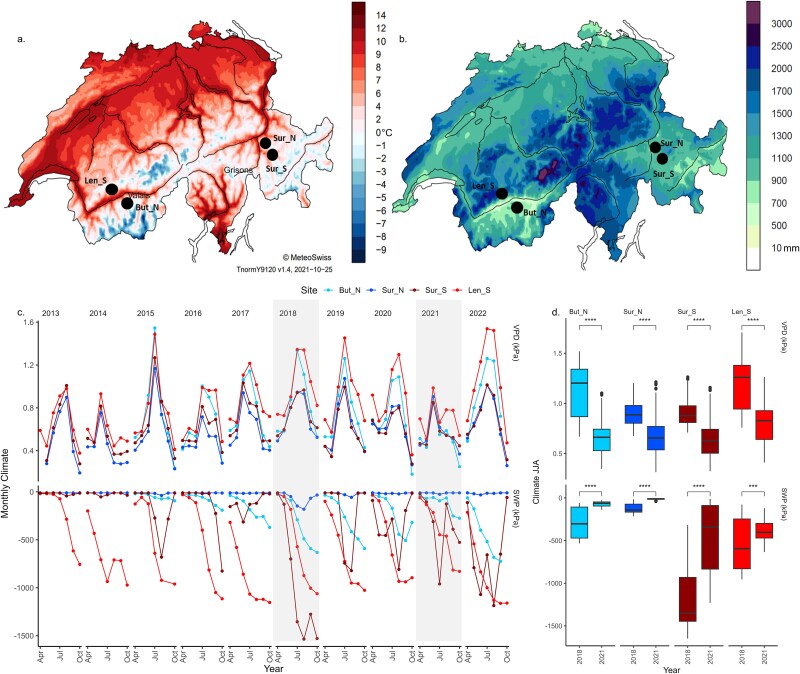
Locations of the sampled sites on maps of (a) mean yearly temperature and (b) mean yearly precipitation (1991–2020, MeteoSwiss). (c) Monthly vapour pressure deficit (kPa) and SWP at 80 cm depth (kPa), smoothed with a 2-week running average for the growing season (April to October) for each year, comparison with temperature and precipitation are shown in Figure S2 available as Supplementary Data at *Tree Physiology* Online. The years 2018 and 2021 are highlighted and used for further comparisons, which combined the lowest SWP and highest VPD conditions for all sites. (d) Comparison of summer VPD and SWP of the two selected years (2018, 2014). Significant differences between pairs are indicated by asterisks (*P* < 0.0001 = ****).

We selected four sites in the Swiss Alps, two in Eastern Grisons (Surava North = Sur_N and Surava South = Sur_S, 1200 m.a.s.l.) and two in Central Valais (Buthan = But_N, 800 m.a.s.l. and Lens = Len_S 1000 m.a.s.l.), as pairs of north and south exposed sites (Figure 2a and b, and Figure S1 and Table S1 available as Supplementary Data at *Tree Physiology* Online). The mean annual temperature ranges from ~6 °C in the Grisons sites to ~10 °C in the Valais sites, and annual precipitation from ~1000 mm in Surava to 600 mm in Valais (1991–2020; MeteoSwiss). In terms of VPD (kPa) and SWP (kPa), the Valais sites are on average warmer and drier than the Grisons sites, with the Lens site being the driest and the Sur_N site the wettest and coolest (Figure 2c, Figure S2 available as Supplementary Data at *Tree Physiology* Online). The years 2018, widely recognized as a major drought year in Central Europe (Gharun et al. 2020), and 2021 are taken as examples of significantly above average dry and wet year respectively at all sites (Figure 2d, Figures S4 and S5 available as Supplementary Data at *Tree Physiology* Online).

We sampled three conifer species, Scots pine (*P. sylvestris,* PISY), Norway spruce (PCAB) and silver fir (*A. alba,* ABAL), in the sites with northern exposition (Sur_N, But_N), whereas only Scots pine was present in the sites on south-facing slopes (Sur_S, Len_S). Scots pine, Norway spruce and silver fir are common coniferous species prevalent in European forests, each playing significant ecological and economic roles. These species exhibit distinct ecophysiological traits and adopt different strategies to withstand environmental stresses, including drought (Vitali et al. 2017, Walthert and Meier 2017, Bose et al. 2020). Scots pine is adapted to a wide range of soil types and environmental conditions, and it occurs on extreme sites where many other tree species are not able to persist (Bigler et al. 2006). It has low hydraulic conductivity and is known for its isohydric water regulation strategy, maintaining a constant leaf water potential and tight stomatal control during dry conditions (Martínez-Vilalta et al. 2009, Walthert et al. 2024). Norway spruce is known for its vulnerability to dry conditions (Vitali et al. 2017) while it also follows an isohydric strategy, minimizing water loss. However, it has been shown to switch to anisohydric behaviour in extreme droughts (Schumann et al. 2024), thus potentially weakening the drought signal of δ^13^C by opening the stomata. Scots pine and Norway spruce have been shown to have similar root systems at Swiss sites (Walthert et al. 2024). On the contrary, silver fir exhibits isohydric behaviour and has a taproot system that provides access to deeper water during dry conditions. Consequently, in general, this species’ transpiration remains largely unaffected under drought (Magh et al. 2020), although trees at sites with a higher soil water storage capacity have been shown to have the highest vulnerability to drought (Nourtier et al. 2014), thus potentially weakening the ^13^C-enrichement connected to drier conditions.

### Environmental data and dendrometer data

Air temperature and relative humidity were measured at hourly time steps using EL-USB-2+ data loggers (Lascar Electronics Ltd, Salisbury, UK). At each of the four study sites, three loggers were mounted on different trees at 2 m height. Values represent the mean of the three loggers. Vapour pressure deficit was calculated following Buck (1981) from temperature and relative humidity. Soil sensors were buried in a soil profile in the central part of each site. In each profile, two to three sensors were embedded at 20 and 80 cm depth (see Figure S2 available as Supplementary Data at *Tree Physiology* Online). Soil water potential and soil temperature were recorded at hourly intervals using MPS-2 dielectric sensors and EM-50 data loggers (Decagon Devices, Pullman, WA, USA). This sensor determines SWP indirectly from the measured water content in its porous ceramic sensor, using dielectric permittivity as a proxy for water content. The SWP measurements were temperature-corrected to 22 °C, necessary to remove erroneous temperature effects from the measurements in dry and cold soils following Walthert and Schleppi (2018).

Stem radius changes at breast height were measured with automatic point dendrometers (ZN11-T-WP, Natkon, Oetwil am See, Switzerland) and logged with LORAWAN nodes (Decentlab GmbH, Dübendorf, Switzerland) on two to three trees per site and species. The data were collected in a central Tree-Net database (Zweifel et al. 2021*a*) and processed into the two fractions of growth and tree water deficit (Zweifel et al. 2016, Knüsel et al. 2021). Cored trees (see Dendrochronological and tree-ring isotope analyses) and trees with dendrometers grew within 20–100 m of the sampled trees.

### Dendrochronological and tree-ring isotope analyses

We took tree cores in October 2022 from five trees per species and site. The removal of resins and other soluble compounds form the cores was achieved through a 24 h Soxhlet extraction with 96% ethanol using a Soxhlet apparatus (standard 500 mL Soxhlet extractor with dimroth condenser, and 1000 mL round flask, e.g., Carl Roth GmbH + Co., Germany; with an electric heater, ELET36002-18, VWR International (Fonti et al. 2025)). A flat surface was produced with a core microtome (Gärtner and Nievergelt 2010). Tree-ring widths (TRWs) were measured for 10 years from 2013 to 2022 at 0.01 mm precision on scanned images with the Skippy system (Gärtner et al. 2024), CooRecorder (Maxwell and Larsson 2021) and cross-dating followed standard procedures (Holmes 1983, Bunn 2010). Tree-ring widths time series (see Figure S2 available as Supplementary Data at *Tree Physiology* Online) showed the largest rings in silver fir in Sur_N (avg. 2.2 mm), followed by But_N (avg. 1 mm), whilst Norway spruce showed similar TRWs at the two sites (avg. 1 mm). Both the rbar and expressed population signal (EPS) values confirmed high consistency among individual trees for all species and sites. Lower values were obtained for Scots pine at the BUT site, where trees exhibited greater variability in dimensions; however, the trees with highest EPS were selected for the intra-annual measurement (see Table S4 available as Supplementary Data at *Tree Physiology* Online). Tree-ring widths were used to determine the positions of the sampling shots of a laser mass spectrometer (LA-IRMS). Within each tree ring, we fixed a resolution of 10 (evenly spaced) shots. The δ^13^C values of each shot might measure a different number of cells depending on cell size due to the fixed shot width, however this is considered sufficient to cover the main intra-annual isotope variations (Pérez-de-Lis et al. 2022). The evenly spaced and tree-ring-width adjusted laser ablation shots ensured that earlywood and latewood were sampled proportionally to their occurrence in each ring, thus minimizing anatomical bias across species and years.

The LA-IRMS system consisted of an isotope-ratio mass-spectrometer to measure the stable carbon isotope ratio δ^13^C (IRMS, HS2022, Sercon, Crewe, UK), a Cryoflex for the collection of produced CO_2_, the ultraviolet laser with a sample chamber and controlling software. An elemental analyser for bulk analysis and comparative analyses was also attached to the IRMS (isoEarth, Sercon). The gases and organic dust from whole-wood cores created by the laser ablation were transported with helium through an oven for combustion to CO_2_, which was collected with liquid nitrogen traps in the Cryoflex. The collected CO_2_ was then released in the IRMS for δ^13^C measurement. During each sample run, we carried out tests with standard cellulose mats of known isotope ratios to determine the memory and precision of the system (±0.3‰). With 10 years of measurements, two sites with three species, two sites with one species and five trees per species, we analysed a total of 400 intra-annual δ^13^C profiles (ca 4000 individual δ^13^C values). We derived mean annual values of δ^13^C in atmospheric CO_2_ from NOAA (http://www.esrl.noaa.gov/gmd) and used them to correct the measured δ^13^C values. The atmospheric data are available as monthly values, but we considered it too speculative to relate these remote data to our site on such a fine scale. To assess the quality of the developed chronologies, we calculated inter-series correlation coefficients (rbar) and the EPS (Wigley et al. 1984).

### Data analysis and statistics

#### Species differences and Day 0 alignment

To test for differential sensitivity to environmental variables at each site and each species, we compared the performance in the wettest and driest years by applying locally estimated scatterplot smoothing (LOESS) to visualize trends in the data and compare patterns across species. Locally estimated scatterplot smoothing was fitted using a span parameter of 0.75 to balance smoothness and data fidelity (Cleveland et al. 2017). This approach allowed us to capture nonlinear patterns while minimizing overfitting. However, to compare between species and years, we found that a normalization of the beginning of activity was necessary. We used automated dendrometer measurements to identify the start of growth dates each year for each site and each species (see Table S1 available as Supplementary Data at *Tree Physiology* Online), designating this ‘Day 0’ as the date of the beginning of the growing season. We accounted for the time lag between the activity start recorded by dendrometers and actual xylem formation. While high-resolution dendrometers allow continuous tracking of stem diameter changes, we recognize that these measurements conflate true radial growth (new cell production) with reversible diurnal and seasonal swelling due to stem hydration, potentially leading to an overestimation of growth onset (Zweifel et al. 2001). However, dendrometers have been validated against microcore sampling and shown to reliably capture growth-onset dates, providing a continuous, non-destructive proxy for cambial phenology (Deslauriers et al. 2007, 2008). This adjustment provided a reliable alignment across different years and species and significantly improved our analyses. Approaches that focus on critical time windows have shown comparative advantages over fixed-length and fixed-time windows (Li et al. 2023). We followed the method proposed by Unterholzner et al. (2024) and constructed a time series aligned by actual growing season that is physiologically relevant and effective in capturing comparably short and variable responses between windows of growth and environmental conditions. We correlated the intra-annual isotope time series with the ‘Day 0’-aligned daily meteorological data. Using this modified time series based on tree functioning periods, rather than fixed calendar periods, we decoupled the environmental variables from calendar dates and translated them into dates preceding or following the start of growth for each year. However, as there are large uncertainties when attempting to identify the end date from dendrometers due to inherent difficulties of assessing an asymptotic curve and due to processes that post-date xylem formation contributing to swelling and shrinkage later in the year (Deslauriers et al. 2007, Zweifel et al. 2021*a*, Etzold et al. 2022), we left the end date open to be matched with the best cross-correlations with VPD and SWP key physiological constraints on late-season assimilation and growth in conifers, and the most significant variables (see Figure S12 available as Supplementary Data at *Tree Physiology* Online).

#### Intra-annual variation of the environmental signal recorded by δ^13^C

To investigate the year-to-year variation in intra-annual δ^13^C curves and their relationship with environmental conditions, we allocated the 10 sequential sectors of 3-week smoothed time series of environmental variables to the 10 consecutive measurements of δ^13^C per year (see Environmental correlations analyses), and based on the knowledge of wood formation speed of conifers (Deslauriers et al. 2007, Etzold et al. 2022, Zweifel et al. 2021*a*). These 10 consecutive windows represented the timing and extent of cell formation in each sector of the tree ring. Following Cuny and Rathgeber (2016), we assigned a shorter length to the windows allocated to the beginning of the growing season and increasing lengths across the ring, albeit assuming no species-specific differences, and we tested different proportions. We then tested the assumption that different shapes of δ^13^C time series correspond to different growing season lengths, as they reflect the recorded environmental conditions (Figure 1). We calculated the cross-correlation between the δ^13^C annual curves and seasonal VPD and SWP aggregates by systematically varying the ‘end date’ of the growing season from 15 June to 30 October. We then selected the optimal ‘end date’ of each δ^13^C-integration (growing) season by identifying the simultaneously highest cross-correlation value between δ^13^C and both, VPD and the lowest one between δ^13^C and SWP. We then identified the optimal growing-season endpoint as the date that maximized the absolute cross-correlation with VPD while simultaneously minimizing the cross-correlation with SWP. This flexibility implies that the correlation window also varied from year-to-year, capturing the inter-annual variations in δ^13^C-integration timing. Once we had identified the optimal δ^13^C-integration window, we investigated the relationship between δ^13^C and the environmental variables at the site level using linear regression models.

#### Environmental correlations analyses

To investigate the overall relationship of δ^13^C with the environmental variables across all years, we calculated running correlations between environmental data and δ^13^C values for each shot for all the sampled trees using the *dendroTools* package (Jevšenak and Levanič 2018) in R (R Core Team 2022). These were carried out using the shot-level δ^13^C data and Day 0 aligned daily meteorological data and applying a moving window approach. We tested different lengths of moving windows to evaluate the relationship between the δ^13^C data and the environmental variables, as described by Jevšenak and Levanič (2018), and found the 3-week smoothing to be the most suitable weighing for noise-to-signal variation. We utilized the non-parametric Kendall’s tau correlation coefficient, which is particularly suited for handling small sample sizes and data that do not necessarily follow a normal distribution (Gibbons and Chakraborti 2010). We performed correlations using 3-week aggregated data for all sites and species. When growing season start data were not accessible, average species data were obtained from the TreeNet monitoring network (Etzold et al. 2019). To evaluate the differences in δ^13^C values for each species and between shots throughout the tree ring, we performed variable analyses of variance, Tukey’s post hoc tests and Bonferroni corrections on absolute values of the correlations. Significance was set at *P* < 0.05.

## Results

### Site- and species-specific intra-annual δ^13^C patterns

**Figure 3:**
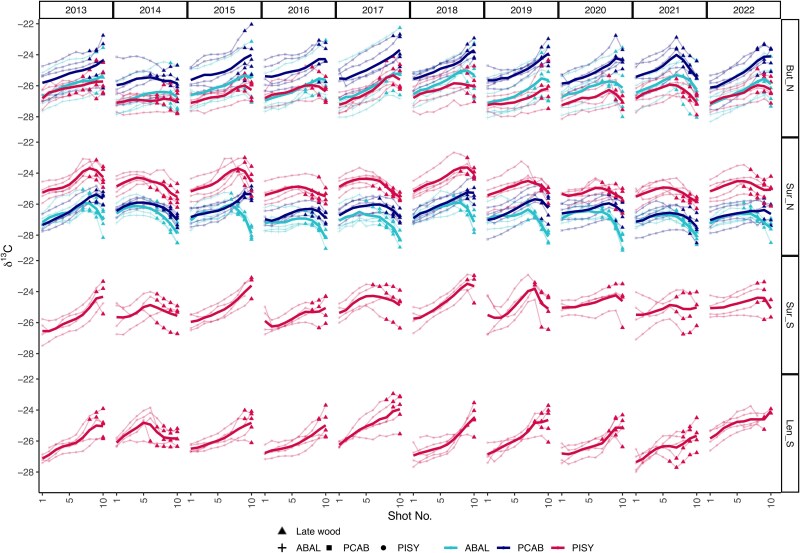
Intra-annual tree-level (thin lines) and average (bold lines) δ^13^C measurements for the years 2013–2022 for the sampled species (*A. alba* (ABAL), *P. abies* (PCAB) and *P. sylvestris* (PISY)), at the respective sites (for details, see Table S1, available as Supplementary Data at Tree Physiology Online). The latewood points have a dedicated shape. The earlywood and latewood boundaries for each tree are shown in Figure S4 available as Supplementary Data at *Tree Physiology* Online. Descriptive statistics for inter-series correlations and the EPS are given in Table S3 available as Supplementary Data at *Tree Physiology* Online.

We investigated the intra-annual tree-ring isotopic variation of silver fir, Norway spruce and Scots pine (respectively ABAL, PCAB and PISY; Figure 3) in the years 2013−2022 by creating high-resolution δ^13^C time series through laser-ablation-IRMS based on 10-shots per ring measurements. The intra-annual δ^13^C time series showed a strong within-species agreement with mean inter-series correlations of 0.6 on average and EPS values of up to 0.88 (see Table S2 available as Supplementary Data at *Tree Physiology* Online). Each species exhibited a clear intra-annual seasonality in the variation of δ^13^C. The three species showed similar trends in δ^13^C values in the first part of the ring but different amplitudes and shapes in the later part of the ring, albeit not strictly attributed to latewood (see Figure S4 available as Supplementary Data at *Tree Physiology* Online). ABAL and PCAB tended to have lower baseline δ^13^C values than PISY in Sur_N, while in But_N PCAB had the highest δ^13^C values (Figure 3, Figure S4 available as Supplementary Data at *Tree Physiology* Online). For PISY, the latewood at the dry sites (Len_S and Sur_S) was narrower than at the moist sites (see Figure S4 available as Supplementary Data at *Tree Physiology* Online).

**Figure 4:**
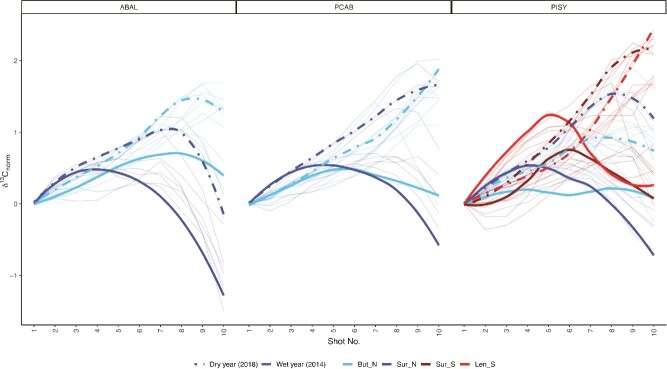
δ^13^C species-specific loess smoothed curves for *A. alba* (ABAL), *P. abies* (PCAB) and *P. sylvestris* (PISY) for the wet (2021) and dry (2018) years. A prominent and consistent bell-shaped and monotonic rise pattern appeared across the four sites. Comparisons across all years are shown in Figure S5 available as Supplementary Data at *Tree Physiology* Online, and comparisons with monthly means are shown in Figure S6 available as Supplementary Data at *Tree Physiology* Online.

When focusing on the very dry year (2018) and wet year (2021), with significantly different VPD and SWP conditions from the mean in June, July and August at all sites, two distinct intra-seasonal patterns emerged (Figure 4): the ‘bell shape’ with low δ^13^C values in the earlywood (shots ~ 1–4), followed by increasing values in the middle stages of the ring, followed by a decline in the latewood (shots ~ 7–10) is associated with moist years; the ‘monotonic raise’ pattern with similarly low δ^13^C values in the earlywood followed by a steep increase and maximum values in the latewood, i.e., lacking the late downward trend can be seen in dry years. The two shapes were common between species at all sites, with two exceptions: at the moist Sur_N site PISY and ABAL preserved the bell shape even in the dry years, and the driest site Len_S, only showed a partial bell shape in wet years (Figure 4).

### Optimal temporal window

**Figure 5:**
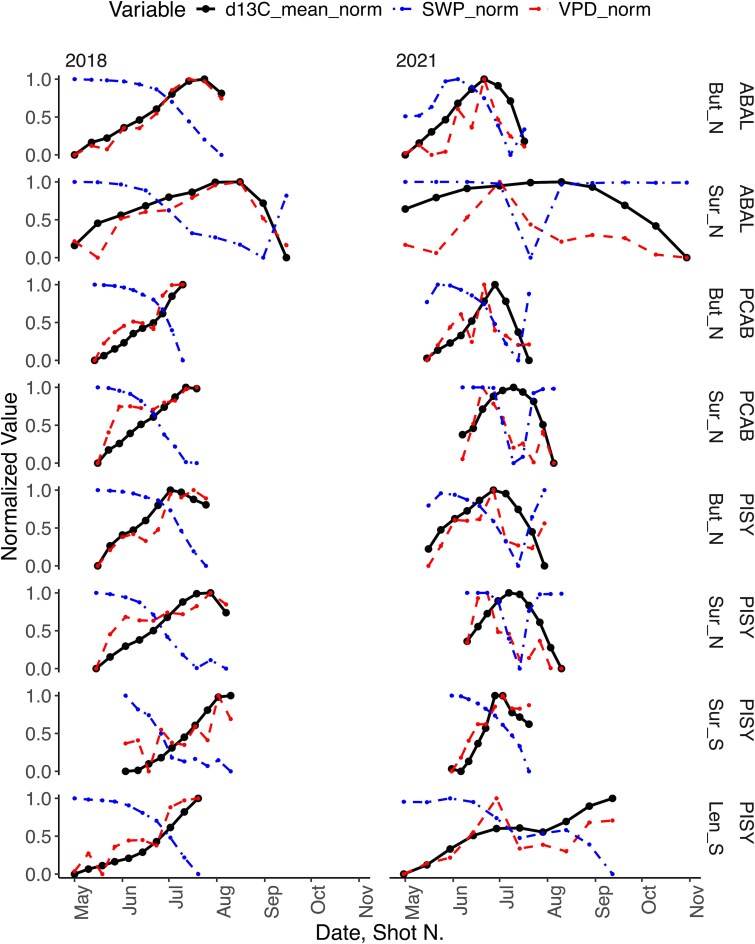
Resulting curves for the normalized environmental variables and δ^13^C time series, restricted to dates between Day 0 and the optimum end date and averaged into 10 time windows aligned to the 10 δ^13^C shots. If multiple maxima occurred, the average date was used. The complete results for cross-correlations, end-date result selection and non-normalized optimized time series for all years are shown in Figures S6 and S7 available as Supplementary Data at *Tree Physiology* Online.

To identify the strongest integration of environmental signal in δ^13^C variation, we identified the temporal window length with the highest cross-correlation results between the intra-annual δ^13^C and the Day 0-anchored environmental variables. In general, δ^13^C followed the VPD trend closely and had a clear inverse relationship to SWP (Figure 5). The maximum correlations occurred mostly in the summer months or later in autumn but showed a strong year-to-year variation (see Figure S6 available as Supplementary Data at *Tree Physiology* Online). Focusing on the dry year 2018, which showed the lowest SWP and highest VPD conditions at all sites, and the wet year 2021, we observed species-specific differences in the identified optimal temporal windows, although the two shapes connected to the dry and wet years remain common across species. We observed negative δ^13^C–SWP correlations, a match with VPD and minimal season-length variation in the drought year of 2018 for all other species and sites (see Figures S6 and S7 available as Supplementary Data at *Tree Physiology* Online), with the exception of ABAL, which consistently showed the latest end dates and δ^13^C bell shapes, indicating that xylem formation extended into the cooler, moister autumn conditions (Figure 5, Figures S6 and S7 available as Supplementary Data at *Tree Physiology* Online). PCAB showed strong year-to-year variation from mid-July to September, with a negative relationship to VPD (Figure 5, Figures S6 and S7 available as Supplementary Data at *Tree Physiology* Online ). PISY in Len_S and Sur_S show sharp monotonic rise pattern linked to a July end date. On the contrary, in the moist year of 2021, most species displayed a bell-shaped δ^13^C curve, with end dates around mid-August. Interestingly, PISY at Sur_S had an earlier end date in 2021, likely due to plummeting SWP in the early season, unlike at other sites where VPD levelled off mid-season. On the contrary, PISY in Len_S showed a strong delay in the maximum cross-correlation in 2021, with a plateau in δ^13^C first emerging in the middle of the ring, followed by a new increase, closely following VPD and SWP patterns, indicating a moist period halfway through the season, followed by a new dry period, thus leading to a new increase in δ^13^C values.

### Correlation of intra-annual tree-ring δ^13^C with SWP and VPD

**Figure 6:**
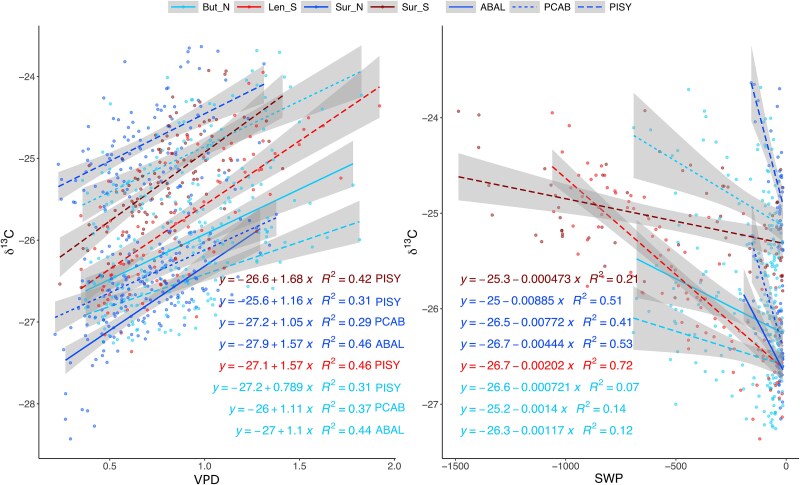
Site and species mean δ^13^C shot values and their relationship with the sequence-dependent averages of VPD and SWP at 80 cm depth, according to the selected window sequence and aligned to Day 0 and end date (the non-aligned correlation can be found in Figure S8 available as Supplementary Data at *Tree Physiology* Online). Linear models were fitted for each species and site. Model equations and explained variance (*R*^2^) are indicated. For SWP, values between −0 and −15 were removed for the model to focus on dry conditions at all sites (all data shown in Figure S9 available as Supplementary Data at *Tree Physiology* Online).

We observed a strong, year-to-year relationship between δ^13^C and the environmental variables, allowing us to quantify the intra-annual consistency of these relations across sites and species. The correlations between tree-ring δ^13^C and both VPD and SWP improved significantly after the alignment to Day 0 and end dates, indicating a more accurate reflection of the incorporated environmental signal in the tree-ring carbon (Figure 6, Figure S8 available as Supplementary Data at *Tree Physiology* Online). Without these alignments, the calendar-dates bound relationships of δ^13^C with the environmental variables were weaker, although the positive correlation of δ^13^C with VPD was partially captured (see Figure S8 available as Supplementary Data at *Tree Physiology* Online). Across all sites and species, there was a positive correlation between VPD and δ^13^C, with the *R*^2^ values ranging from 0.29 to 0.46 (Figure 6). The sites Len_S and Sur_N display the steepest slopes (0.145 and 0.142, respectively), suggesting a strong control of VPD on δ^13^C at these locations. Conversely, a negative correlation was observed between SWP and δ^13^C. As SWP became more negative (indicating drier soil conditions), δ^13^C values tended to become less negative. The *R*^2^ values were generally higher for the south-exposed sites and ranged widely, from 0.07 for ABAL in BUT_N to 0.72 for PISY in Len_S. The strong correlation of PISY at Len_S points to the substantial influence of SWP on δ^13^C at this site. The moist site of Sur_N showed the steepest negative slope, where a small change to lower SWP led to a relatively large increase in δ^13^C, albeit at higher absolute values than at the drier site. These findings underscore the influence of local site conditions and species-specific characteristics on δ^13^C values in response to VPD and SWP, but also the strong general trends that become apparent once the timing of xylem formation has been identified.

### Day 0 aligned correlation analysis and shot-level climate signal

**Figure 7:**
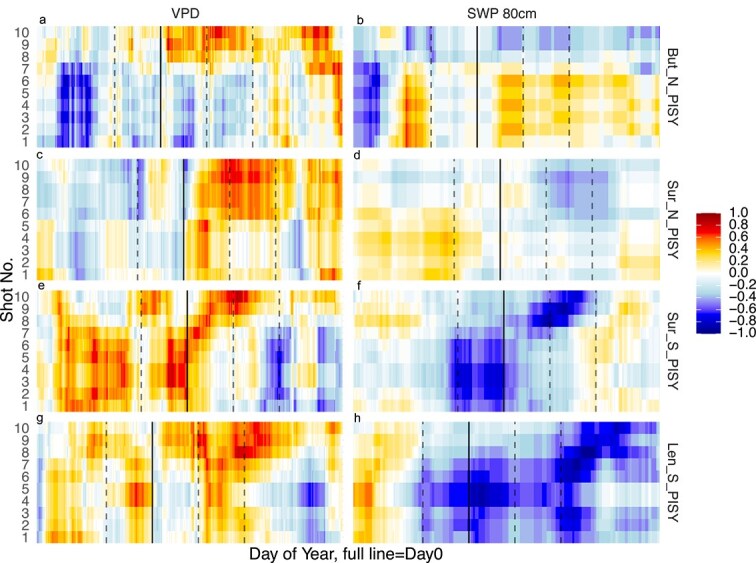
Heatmaps displaying Kendall’s tau correlation coefficients between δ^13^C values measured in the 10 consecutive shots in tree-rings (*y*-axis) and VPD (left panels, a, c, e, g) and SWP at 80 cm depth (SWP 80 cm, right panels, b, d, f, h), aggregated into 20-day moving windows for *P. sylvestris* across all sites. The *x*-axis represents the day of the year, with solid vertical lines indicating Day 0 and dashed lines indicating 50 days before and 50 and 100 days after Day 0. The staggering of the solid line across the panels reflects the difference in the start date for growth among the species at each site. All species responses are in Figure S11 available as Supplementary Data at *Tree Physiology* Online.

The strength of high-resolution measurement of δ^13^C is the access to cell-level measurements along the ring. Thus, traditional correlation of tree-ring variations with environmental variables can be performed for each shot. We evaluated the recorded signal in δ^13^C intra-annual variation for the measured 10 years, through a moving window correlations approach, by aligning each year to the beginning of growth activity (Day 0 alignment is indicated by the solid line in Figure 7, all sites in Figure S10 available as Supplementary Data at *Tree Physiology* Online). We calculated the δ^13^C correlations with environmental data for each measured shot for the on-site measured variables (temperature, air humidity, soil moisture and soil temperature, Figure S11 available as Supplementary Data at *Tree Physiology* Online), and tested the common approach of using calendar-based datasets (see Figure S12 available as Supplementary Data at *Tree Physiology* Online), however, the correlations with Day 0 alignment were significantly stronger. Consistently positive correlation patterns during the mid- to late-growing season appear between δ^13^C and VPD for most species, with an overall median correlation of 0.6, and consistently negative correlation patterns emerged between δ^13^C and SWP, with an overall median correlation of −0.4. On average, shots showed similar correlations in groups of three for both variables. The major differences in the shot-level correlations can be seen in shots 8–10, which included mostly latewood material (see Figures S13 and S14, Table S4 available as Supplementary Data at *Tree Physiology* Online). PISY, occurring at all four sites, thus covering the largest environmental gradient, represents the main visible trend along the shots: one with a sign change in the correlations in the last two shots of the ring (Figure 7a and b, But_N); the other with a gradual temporal shift of highest correlations from early to late shots (Figure 7e−h, in the dry sites of Sur_S and Len_S), or, finally, a general lack of strong correlations trends (Figure 7c, Sur_N).

## Discussion

### Common intra-annual tree-ring δ^13^C patterns between conifer species, dependent on wet and dry seasonal patterns

In this study, we investigated the intra-annual δ^13^C patterns in tree rings for silver fir, Norway spruce and Scots pine in two pairs of sites in two Swiss regions over a 10-year period to assess the environmental and physiological signals stored in wood isotopic ratios. Using a multi-species approach at locations varying in water availability enabled us to tease apart the dominant environmental factors and identify the confounding effect of species and site on δ^13^C–environmental patterns. Although distinct species-specific differences could be shown in the average rates of δ^13^C (Figure 3, Figure S4 available as Supplementary Data at *Tree Physiology* Online), common intra-annual patterns across species and sites were linked to atmospheric and soil dryness. We observed a strong agreement in intra-annual δ^13^C within sites and between species, especially in the wettest conditions (see Tables S2 and S3 available as Supplementary Data at *Tree Physiology* Online), in agreement with Hp1. This consistency is an important prerequisite for the widespread application of high-resolution isotope analyses to better understand the effects of drought on forest trees.

The wet year and north-facing sites showed most consistently a bell-shaped δ^13^C pattern, as water limitation is rare (Figure 4), species differences in stomatal regulation are minimized (Schumann et al. 2024) and the cambial activity is more synchronized (Cuny et al. 2012). This is consistent with findings of other high-resolution intra-annual investigations of Scots pine in Scandinavia (e.g., Soudant et al. 2016, Tang et al. 2022, Rinne-Garmston et al. 2023), for Norway spruce in another intra-annual study at a cool and moist alpine site (Martínez-Sancho et al. 2022). All three species in the moist site of Sur_N show the highest growth rates (see Figure S2 available as Supplementary Data at *Tree Physiology* Online) and a common bell shape (Figures 3 and 5), suggesting a strong overlap of the canopy functioning of the three species, a clear shared environmental signal recorded, and no strong water limitation effects. Where differences in the intra-annual δ^13^C pattern did occur, this may be attributable to differences in the time span of stem growth activity (Etzold et al. 2019). Scots pine and spruce show a good season-integration match in mid-August, while silver fir showed a stronger decline in δ^13^C in the later part of the ring (Figure 5), suggesting that the carbon used in the latewood is formed under cool and moist conditions, likely reflecting xylem production and lignification extending later in the autumn period. The 2‰ higher average δ^13^C in the later shots in pine and spruce further suggests the use of carbon formed in warmer and drier conditions for the formation of that woody tissue, and thus an earlier cessation of xylem formation compared with silver fir (Figures 3 and 4).

The wet years showed the common bell shape, while in dry years, the intra-annual pattern changed drastically from a bell shape to a monotonic rise pattern, i.e., the maximum isotope values were found in the latest cells (Figures 4 and 5). Under drought, trees minimize water loss via stomatal closure, resulting in ^13^C-enriched assimilates (McCarroll and Loader 2004, Gessler et al. 2009). Simultaneously, cambial downregulation slows cell division and wall formation, reducing the sink strength for new assimilates (Hommel et al. 2016). Additionally, phloem flow is restricted, limiting the transport of enriched sugars to the cambium (Winkler and Oberhuber 2017). This leads to diminished incorporation of fresh photosynthates in latewood, or even a complete shift to stored reserves or a complete arrest of growth (Rathgeber 2017). Thus, when VPD is highest and SWP is critically low like in 2018, the trees experience rapid and more severe stomatal responses and stomatal closure earlier in the growing season (Grossiord et al. 2020, Petek-Petrik et al. 2023), truncating the carbon assimilation period (Pflug et al. 2015), which together with turgor limitation, results in the cessation of production of new cells (Cabon et al. 2020, Mitchell et al. 2014, Peters et al. 2021, Zweifel et al. 2021*b*), resulting in the observed monotonic rise pattern (Figure 4). The year 2018 is a well-known drought year in the study region that negatively affected forest productivity by 20% in Switzerland (Gharun et al. 2020), thus supporting our assumption of an early stop of xylem production. However, silver fir is the only species not showing a monotonic rise pattern in 2018 at the Sur_N site, indicating only a minor impact of this drought year on this species. This observed dependency of inter-annual variation in δ^13^C on wet and dry conditions aligns with our conceptual diagram and Hp2, showing the direct interplay between soil and atmospheric dryness converging to create distinct δ^13^C patterns within annual tree rings in conifer trees.

In our case, the strong agreement of δ^13^C curves between trees of the same species supports the environmental-driven δ^13^C signal in conifers. As earlywood production in conifers is influenced by stored compounds only to a minor degree (Dickmann and Kozlowski 1970, Barbour et al. 2002), the δ^13^C variations may better reflect current growing season conditions than in deciduous species (Monson et al. 2018*a*, 2018*b*). This common pattern appears to be distinctly different from that of broadleaf deciduous trees, which have been shown to have a seasonally recurring tri-phase carbon isotope pattern, with: (i) the highest values of δ^13^C in the earlywood (of up to 5‰ higher), followed by (ii) a decline and plateau, with minimum δ^13^C values occurring in the latewood and (iii) a gradual increase of δ^13^C values at the very end of each tree ring, linked to changes in downstream processes of carbohydrate metabolism, and strongly connected to the shifts in heterotrophic and autotrophic mechanisms that support wood formation (Helle and Schleser 2004, Offermann et al. 2011).

### Finding the activity window and limiting factors: VPD drives the intra-annual δ^13^C variation, and SWP triggers growth cessation

One of the challenges in the interpretation of intra-annual δ^13^C values is relating them to the timing of growth, which varies from year-to-year. However, here we show that isotope patterns can, in fact, help us to infer growth dynamics through the recording of environmental stressors on tree physiology. The relationship between δ^13^C and VPD and SWP, after alignment to the Day 0 and end dates (Figure 6), shows significantly improved correlations with δ^13^C values for both VPD and SWP, thus providing the opportunity to connect environmental variables with δ^13^C variations. Other research has shown that deriving an optimal period of correlation between δ^13^C values and local environmental data leads to a more accurate prediction of intra-annual variation in tree responses (Zhang et al. 2012, Soudant et al. 2016, Castagneri et al. 2017, Ziaco 2020). Soudant et al. (2016) showed that an optimized time-frame approach based on the timing of key phenological stages, such as cell formation, improved the linear regressions between δ^13^C and local environmental variables (i.e., light availability in Scandinavia). Thus, by accounting for statistically optimized periods, we not only improve the predictive power of δ^13^C variation on both intra- and inter-annual scales but also provide a direct time–space link in the wood structure and quantify the effective contribution of the environmental variables to the δ^13^C variation, paving the way to a more mechanistic understanding of tree-ring carbon deposition.

A consistent positive relationship of VPD with δ^13^C was shown regardless of species and position within the tree ring, thus supporting the link between canopy functioning and seasonal variation in δ^13^C in the xylem (Figure 6). This is in accordance with the findings by Zweifel et al. (2021) based on dendrometer-measured stem growth data at the same sites. The SWP relationship with δ^13^C was more site-dependent (Figure 6). The differences in intercepts and ranges do indicate species-specific adaptations like isohydricity, or different niche rooting systems; however, the overall pattern remains consistent. The moist site of Sur_N showed the steepest negative slope, indicating that a small change of lower SWP leads to a relatively large increase in δ^13^C. This suggests that it is not the absolute drop in SWP that can lead to the interruption of xylem formation but rather the site-relative drop. This was apparent in the 2018 drought when a monotonic rise pattern occurred in all sites, including the wettest site of Sur_N (but for ABAL at Sur_N, Figure S8 available as Supplementary Data at *Tree Physiology* Online). There we observed significantly lower VPD and higher SWP than at all the other sites; while all sites commonly reached values of –1000 kPa, the minimum SWP in Sur_N reached in 2018 was −150 kPa at 80 cm depth, and −700 kPa at 20 cm depth (Figure 1, Figure S1 available as Supplementary Data at *Tree Physiology* Online). In the other years, SWP at Sur_N generally only reached a minimum of −40 kPa at 80 cm and −300 kPa at 20 cm depth, suggesting uncharacteristically dry conditions of the 2018 growing season leading to an early end of xylem production. Meanwhile, we observed bell-shaped curves in other years (e.g., 2019, 2022), even though VPD was higher than in 2018, but SWP was not lower. Notably, Sur_N is a relatively cold site; thus, the rooting depth and root density could be reduced due to the characteristic low soil temperatures. Therefore, already a slight drop in SWP could have a relatively high negative impact on growth because of a relatively strong reduction of water uptake.

Overall, our results suggest that although VPD drives the seasonal variation in δ^13^C in tree rings, by influencing stomatal closure and photosynthetic rates, the cessation of growth is connected to the synchronization of atmospheric and below-ground dryness (Etzold et al. 2022), with soil water availability as the factor that can induce an abrupt stop in xylem production. If SWP is not significantly lower than in other years, trees are acclimated to the site conditions and can carry on with growth until autumn, even at high VPD (Etzold et al. 2019), provided that enough soil water is available for stem growth. Thus, we conclude that by identifying the timing of the growth period, we can identify not only the environmental drivers of δ^13^C variation (Hp3) but also the potential trigger of early growth cessation at both dry and moist sites. This cross-site consistency suggests a generalizable pattern in conifer drought physiology, where VPD primarily modulates isotopic discrimination and SWP governs growth cessation timing, modulated by rooting depth and soil moisture buffering capacity.

### Within-ring δ^13^C variation reflects temporal shifts in environmental signal integration

Environmental information stored in tree rings changes from early to latewood reflect, on one hand, the seasonal changes in the factors driving cell production (Castagneri et al. 2017) and, on the other hand, the lag in the integration of canopy assimilates into the xylem tissue, which depends on the stage of growth during the growing season (Gessler et al. 2009, Cuny et al. 2014*b*). High-resolution measurements of δ^13^C allowed us to identify intra-annual changes in seasonal signal integration along tree rings, with significant differences between the first and last part of the ring and significant changes at the fourth and eighth shots (see Figures S13 and S14 available as Supplementary Data at *Tree Physiology* Online). These points of transition represent critical periods in wood formation, when the trees are particularly sensitive to environmental changes, supporting our hypothesis that the strength of the correlation between δ^13^C environmental variables differs across the ring (Hp3). The strength of the δ^13^C correlations with environmental conditions is dependent upon both the physiological responses of photosynthetic and post-photosynthetic carbon isotope discrimination, but also on the amount of living tissue at each time point (Soudant et al. 2016, Wieloch et al. 2024). It has been shown that a large number of wood cells are usually formed simultaneously in early summer, which is consistent with the findings that the critical period for maximum rates of cell division occurs around the summer solstice in temperate and boreal forests (Balducci et al. 2016, Carrer et al. 2017, Gričar et al. 2022). These periods of rapid cell division result in a heightened sensitivity to summer atmospheric conditions, as also shown in whole-ring width series (Carrer et al. 2017, 2018, Belokopytova et al. 2019, Zelenov et al. 2024). In annually resolved isotope records, this could lead to the amplification of early summer signals and the attenuation of late-season signals, especially under stressful conditions where cambial activity may go undetected (Pflug et al. 2015, Pérez-de-Lis et al. 2022). The distinction between early and latewood is a good first estimation of the intra-annual variation in carbon assimilation (see Figure S3b available as Supplementary Data at *Tree Physiology* Online). For example, Norway spruce showed the highest annual δ^13^C values in But_N, while Scots Pine was the one with the highest values in Sur_N, identifying potential acclimation differences between the sites. However, through high-resolution interannual analyses, such as in this study, we can identify the multiple shifts in the strength and direction of the environmental signals throughout the growth year. For example, silver fir exhibited a pronounced bell-shaped intra-annual δ^13^C pattern even under drought conditions, whereas Scots pine and Norway spruce shifted to a monotonic-rise pattern in δ^13^C values. This difference can be partly attributed to the silver fir’s deeper and more extensive root system, which allows sustained access to subsoil moisture through mid-season (Brunner et al. 2015). By contrast, Norway spruce, which concentrates roots in shallower soil horizons, experiences more rapid declines in water uptake as surface layers dry (Puhe 2003). Moreover, silver fir displays a relatively anisohydric stomatal strategy, delaying closure despite rising VPD, which prolongs discrimination against ^13^C into the late summer (Peters et al. 2019). Scots pine at the But_N site showed a switch from negative to positive correlations between δ^13^C values and environmental variables in the 8–10th shots for all species (Figure 7a and e). These shots captured latewood material, and the substantial differences in δ^13^C values may be due to the transition from growth limiting to growth-promoting effects of warmer temperatures during the later stages of the growing season. On the contrary, at the dry sites Scots pine showed a distinct temporal link of the δ^13^C correlation with VPD and SWP, where the shot-level strongest correlations followed the advancement of the growing season, producing a clear ‘diagonal’ of maximum correlations (Figure 7e−h). This underscores its sensitivity to VPD and SWP, but also its reliance on fresh assimilates that can record seasonal variations (Barbour et al. 2002, Kress et al. 2009). As such, Scots pine is a highly promising species to record intra-annual variation in VPD and SWP.

In this study, we introduce a novel, integrated approach that combines high-resolution carbon isotope analysis with daily climate correlation (Jevšenak 2019). This framework enables us to resolve intra-annual isotope variations and to pinpoint the timing of seasonal climatic influences. We recognize, however, that our 10-year dataset, while substantial in raw data volume, represents a relatively short series for correlation analysis, which can lead to inflated coefficients (Torbenson et al. 2024). To mitigate this, we employed the more conservative Kendall’s tau metric. Moreover, the detected climate–carbon relationships align with the expected seasonal windows, lending confidence to our results. Finally, because of the limited time span, we did not assess potential carry-over effects, which could further clarify the dynamics of climate–carbon interactions (Andreu-Hayles et al. 2022).

## Conclusions

By understanding the relationship between environmental variability and δ^13^C variation at a high resolution, we enhanced the interpretation of information stored within tree rings and highlighted the direct temporal link between wood formation and growing conditions. While we find a very strong and consistent VPD signal across all sites and species, we also show that SWP plays a key role in the interruption of growth processes, regardless of the minimum values reached (i.e., a small relative drop in SWP at a moist site has a more dramatic impact on the δ^13^C signal than recurring dry conditions at a drier site). These findings help in understanding the effects of soil drought and VPD on trees under increasing climate change impacts. This understanding may furthermore be useful to inform ecosystem-level carbon sequestration models based on future climate scenarios of potential thresholds in soil or air dryness that hinder growth and thus limit carbon sequestration. Meanwhile, the consistent VPD signal across species and sites offers high potential for reconstructions of summer conditions. Scots pine, in particular, shows high potential, owing to its large distribution and strong connection to current-year assimilates, although further research is needed to investigate the consistency of this signal across its full range of occurrence. Finally, high-resolution intra-annual changes in δ^13^C can be particularly useful on sites and in times with no access to instrumental data as δ^13^C patterns show strong commonality across sites and reflect the combined impact of atmospheric and soil dryness. Further comparisons between various temporal and spatial scales, but also across species and stand dynamics, are now needed to deepen our understanding of how trees respond to climate change and how they incorporate physiological responses to environmental conditions in their tree rings.

## Supplementary Material

Appendix_review_ForResubmission_tpaf120

## Data Availability

The data and code that support the findings of this study are openly available in ENVIDAT at https://www.doi.org/10.16904/envidat.564.
