# Supplementary material for: Effects of soil and atmospheric drought on intra-annual δ13C patterns in tree rings
Source: Tree Physiol. 2025 Sep 27;45(11):tpaf120. doi: 10.1093/treephys/tpaf120 (PMC12596289; doi:10.1093/treephys/tpaf120)
Supplement: Appendix_review_ForResubmission_tpaf120 [file appendix_review_forresubmission_tpaf120.docx]

**Appendix**

Fig.S. 1 Map of sampled sites across Switzerland, two in Eastern Grisons (Surava North = Sur_N and Surava South = Sur_S,) and two in Central Valais (Buthan= But_N and Lens=Len_S), as pairs of north and south exposed sites (map.geo.admin.ch).

Table S. 1 Geographic characteristics of the study sites. The table provides the site name, site ID, exposition (North or South facing), geographic coordinates (longitude and latitude, WGS 84), and elevation (in meters above sea level) for the selected sites.

| **Site** | **Site ID** | **Exposition** | **Longitude** | **Latitude** | **Elevation** |
| --- | --- | --- | --- | --- | --- |
| Lens | Len_S | South | 7.43543001 | 46.2677184 | 1000 |
| Surava | Sur_S | South | 9.62282043 | 46.6740728 | 1200 |
| Surava | Sur_N | North | 9.623784 | 46.6749437 | 1200 |
| Buthan | But_N | North | 7.62880456 | 46.2932722 | 800 |


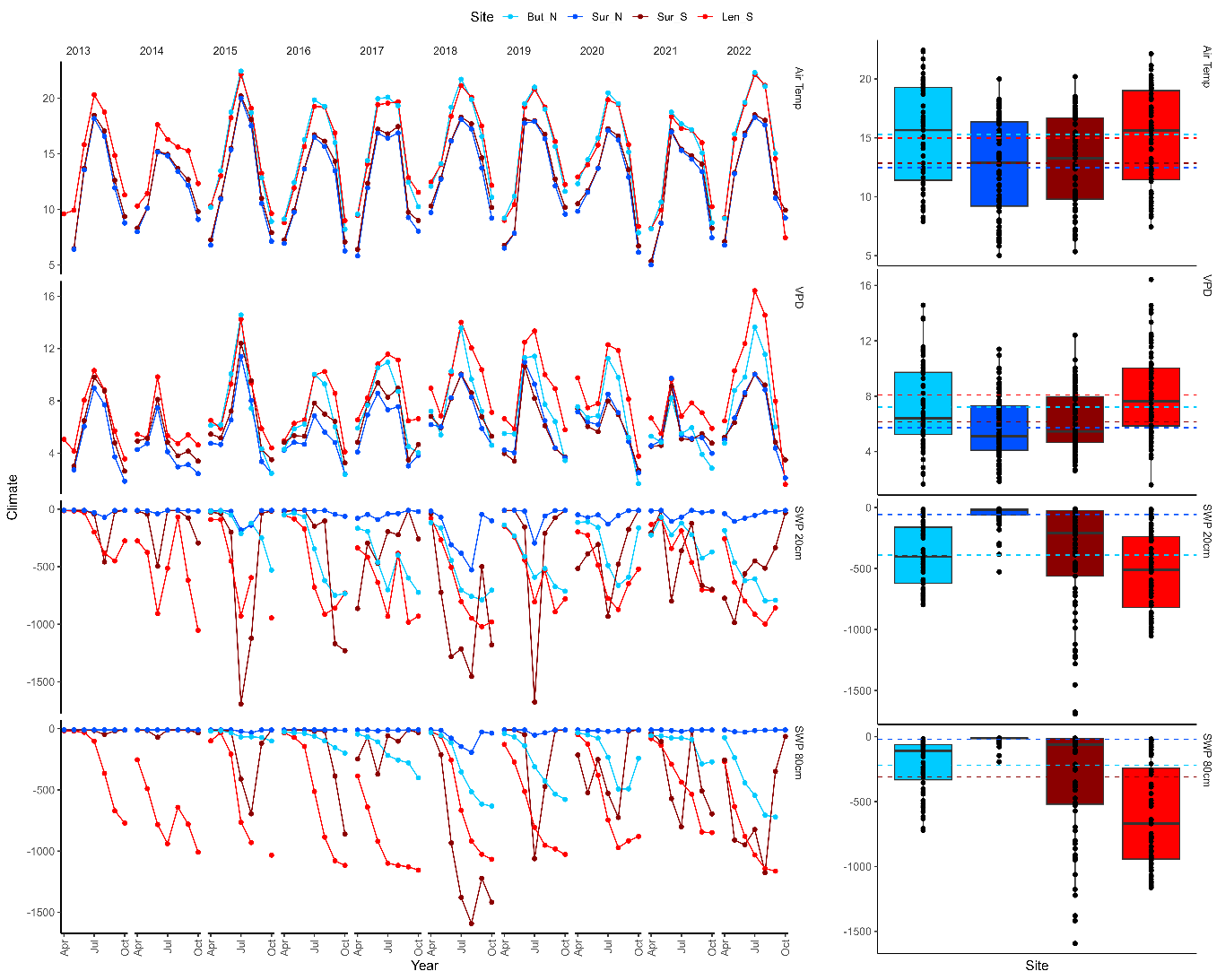


Fig.S.2 (a) Monthly environmental variables (air temperature (Air Temp, °C), vapour pressure deficit (VPD, kPa), soil water potential (SWP, kPa) at 80 cm and 20 cm depth) at each site, smoothed with a two-week running average for the growing season (April to October) for each year. (b) Boxplots and significant differences in climate parameters among the sites in the period of May to August for the years 2013 to 2022 (P≥0.05 = not significant (ns); P<0.05 = *; P<0.01 = **, P<0.0001 = ****).

Table S. 2 Start dates derived from dendrometer data for each year, site and species, used for the Day0 correction.

| **Site and Species** | **2013** | **2014** | **2015** | **2016** | **2017** | **2018** | **2019** | **2020** | **2021** | **2022** |
| --- | --- | --- | --- | --- | --- | --- | --- | --- | --- | --- |
| **But_N** |  |  |  |  |  |  |  |  |  |  |
| **ABAL** | 120 | 120 | 120 | 120 | 120 | 120 | 120 | 120 | 120 | 120 |
| **PCAB** | 135 | 135 | 134 | 134 | 134 | 134 | 153 | 123 | 134 | 135 |
| **PISY** | 130 | 130 | 135 | 135 | 135 | 135 | 135 | 135 | 135 | 135 |
| **Len_S** |  |  |  |  |  |  |  |  |  |  |
| **PISY** | 116 | 116 | 115 | 106 | 108 | 121 | 123 | 112 | 119 | 126 |
| **Sur_N** |  |  |  |  |  |  |  |  |  |  |
| **ABAL** | 120 | 120 | 120 | 120 | 120 | 120 | 120 | 120 | 120 | 120 |
| **PCAB** | 146 | 145 | 145 | 145 | 145 | 135 | 159 | 146 | 157 | 140 |
| **PISY** | 139 | 140 | 126 | 133 | 129 | 135 | 148 | 139 | 161 | 146 |
| **Sur_S** |  |  |  |  |  |  |  |  |  |  |
| **PISY** | 151 | 150 | 141 | 140 | 153 | 153 | 155 | 145 | 160 | 165 |

| Site & Species | Mean TRW | rbar | eps |
| --- | --- | --- | --- |
| BUT_ABAL | 1.04 | 0.403 | 0.802 |
| BUT_PCAB | 1.08 | 0.551 | 0.83 |
| BUT_PISY | 0.545 | 0.149 | 0.611 |
| SURN_ABAL | 2.31 | 0.409 | 0.776 |
| SURN_PCAB | 1.04 | 0.368 | 0.744 |
| SURN_PISY | 0.808 | 0.303 | 0.723 |
| SURS_PISY | 0.682 | 0.586 | 0.876 |
| LEN_PISY | 0.747 | 0.598 | 0.881 |

Table S.3 Descriptive statistics for tree-ring chronologies. Mean tree-ring width (TRW) between 2013 and 2022, and the inter-series correlation coefficient (rbar) and expressed population signal (eps) values for the five trees sampled for each site and species.

.

| *Site & Species* | **n.cores** | **n.trees** | **n** | **n.tot** | **rbar** | **eps** | **snr** |
| --- | --- | --- | --- | --- | --- | --- | --- |
| *But_N_ABAL* | 5 | 5 | 5 | 10 | 0.588 | 0.877 | 7.147 |
| *But_N_PCAB* | 5 | 5 | 5 | 10 | 0.689 | 0.917 | 11.065 |
| *But_N_PISY* | 5 | 5 | 5 | 10 | 0.61 | 0.887 | 7.819 |
| *Sur_N_PCAB* | 5 | 5 | 5 | 10 | 0.597 | 0.881 | 7.407 |
| *Sur_N_ABAL* | 5 | 5 | 5 | 10 | 0.679 | 0.914 | 10.582 |
| *Sur_N_PISY* | 5 | 5 | 5 | 10 | 0.553 | 0.861 | 6.185 |
| *Sur_S_PISY* | 5 | 5 | 5 | 10 | 0.588 | 0.877 | 7.147 |
| *Len_S_PISY* | 5 | 5 | 5 | 10 | 0.684 | 0.916 | 10.835 |

Table S.4 Descriptive statistics, inter-series correlation coefficient (rbar) and expressed population signal (eps) for the five trees sampled for δ^13^C chronologies for each site and species.


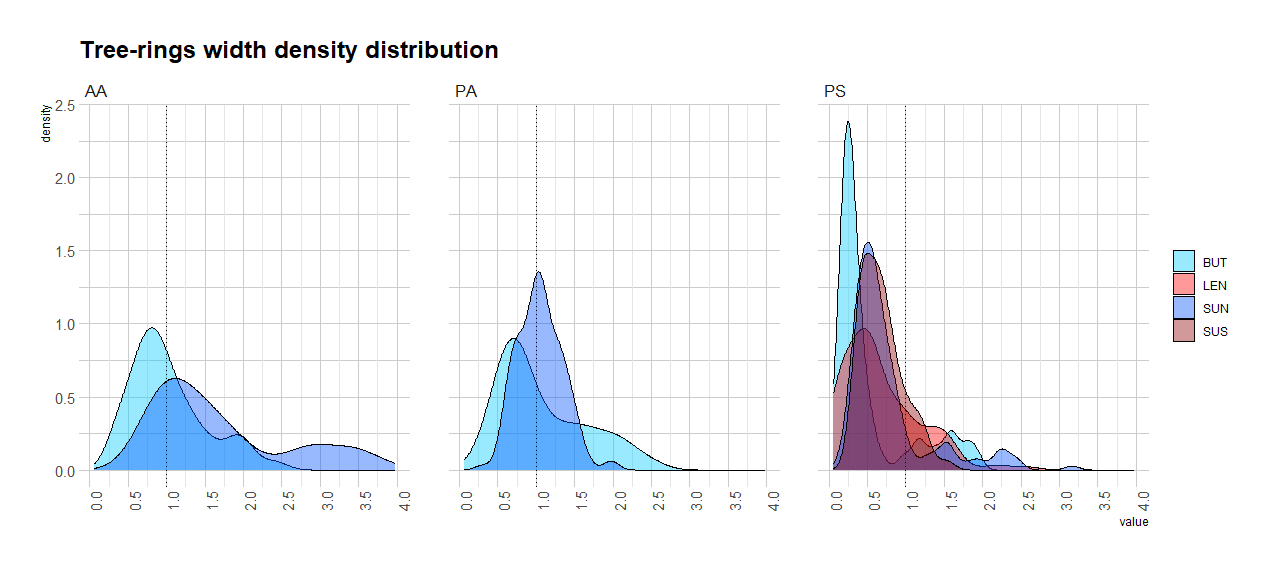

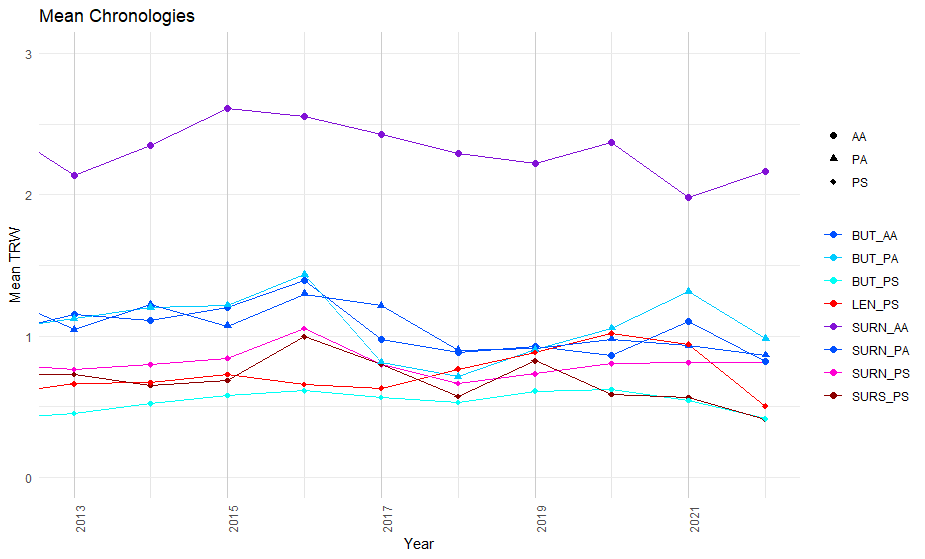


a.

b.

Fig.S.3 (a) Mean chronologies of tree-ring width (TRW) for each species (AA=ABAL, PA= PCAB, PS=PISY) and site, and (b) their mean distribution curves.


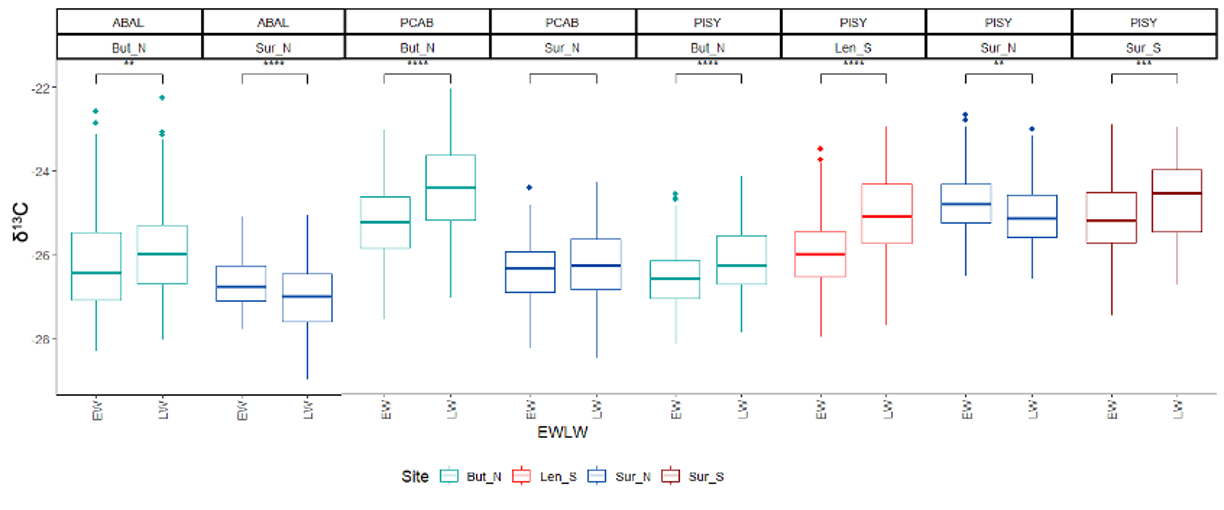

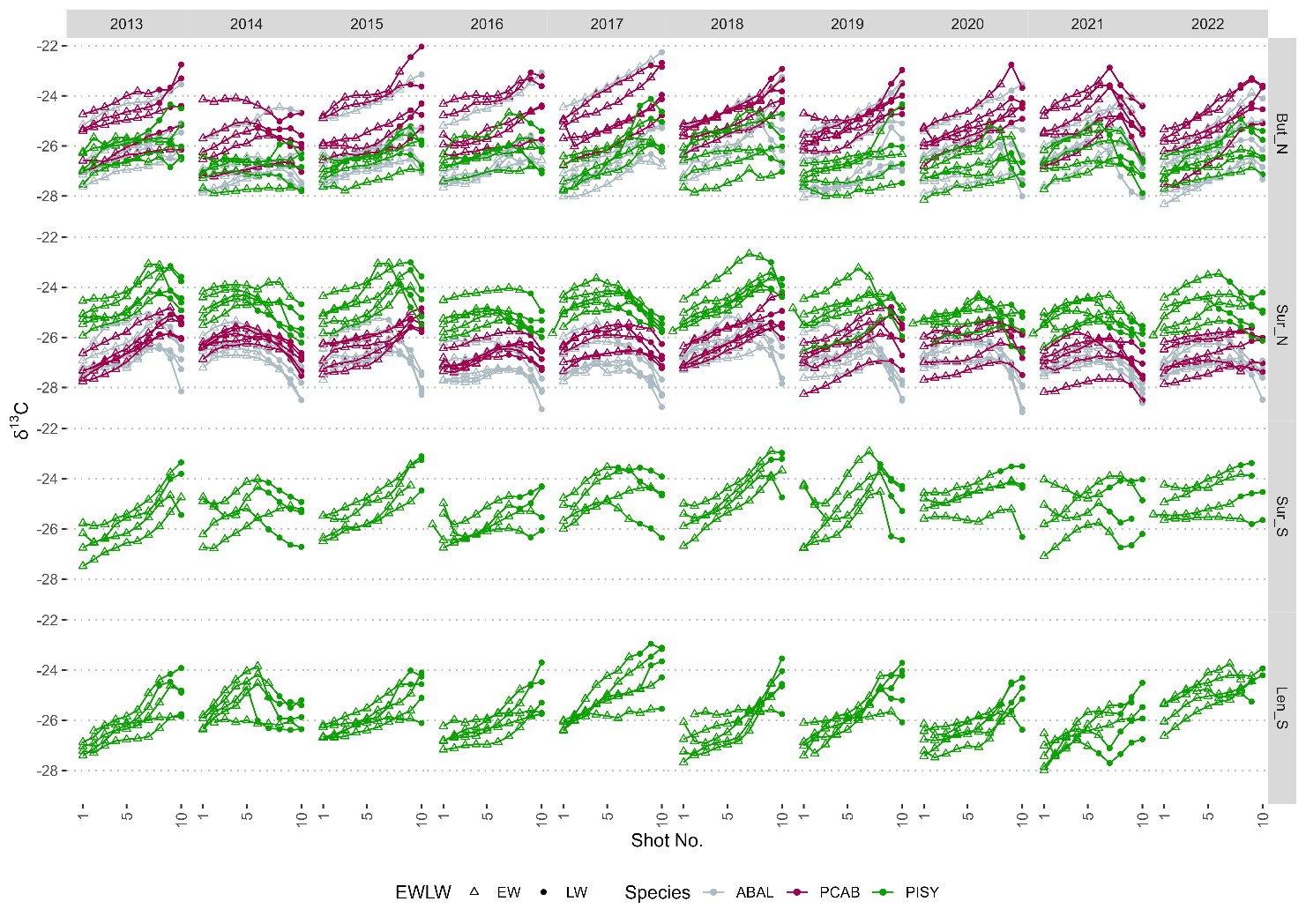


Fig.S.4 a. Intra-annual tree-level δ^13^C measurements for the years 2013-2022 for the sampled species (Abies alba (ABAL), Picea abies (PCAB), and Pinus sylvestris (PISY)), at the respective sites (for details see Table1). Shots attributed to earlywood ) and latewood (LW) are indicated with different symbol shapes. b. differences in EW and LW between early and late wood for all years, at each site and for each species.


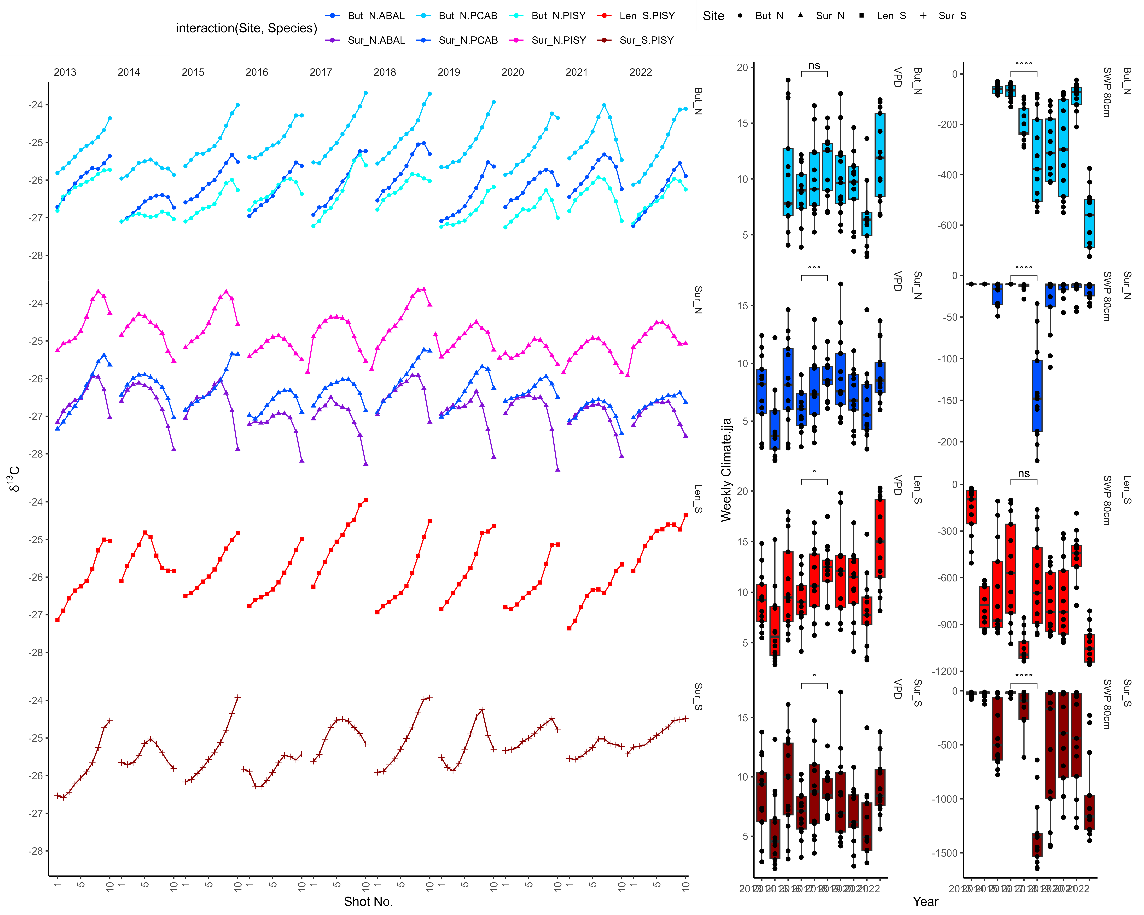


Fig.S.5 δ^13^C inter-annual species averages curves for all years. b. Summer Vapor Pressure Deficit (VPD) and Soil Water Potential at 80 cm depth (SWP 80cm), please note the variable y scales when comparing across sites. Significant differences between pairs are indicated by asterisks (P<0.001=***, P<0.0001=****).


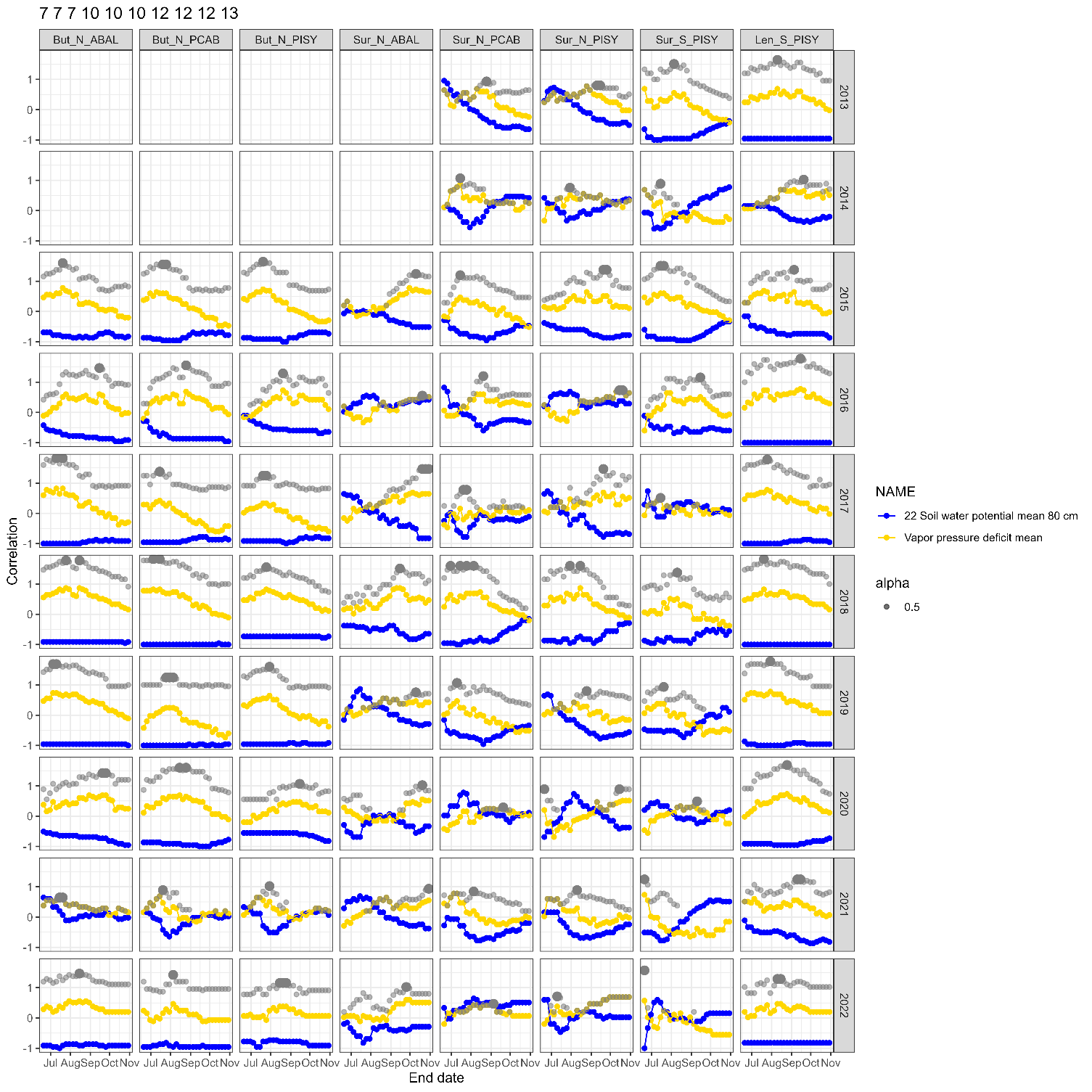


Fig.S.6 Results of the cross-correlation analyses for all years, and the identified maximum correlation dates indicated by the gray dot, in the period between June and November, used for the identification of the optimal xylem production period.

Fig.S.7 Normalized vapour pressure deficit (VPD) and soil water potential (SWP), aligned to Day0, and the most significant cross-correlation values, averaged to 10 windows allocated to each tree-ring shot


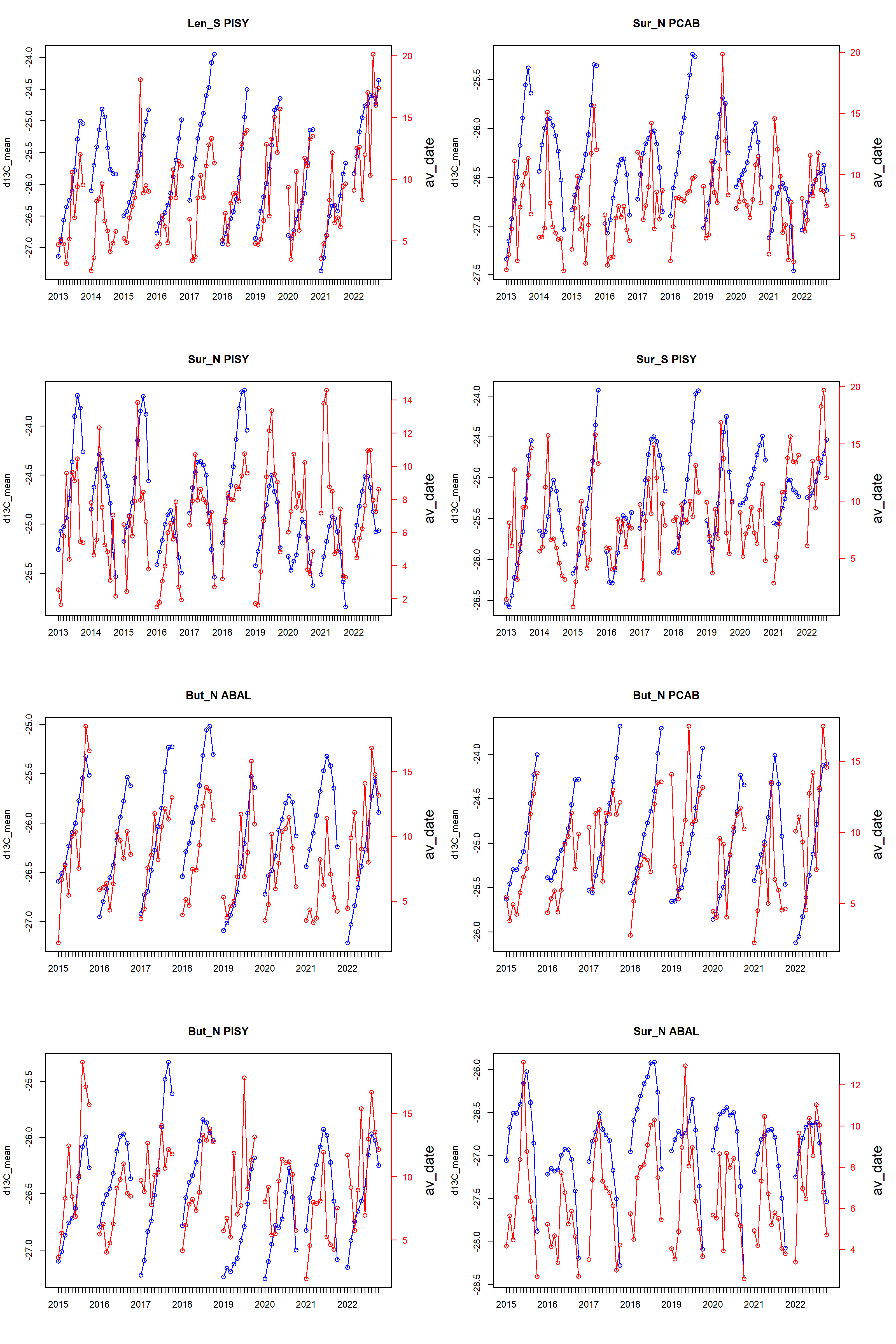


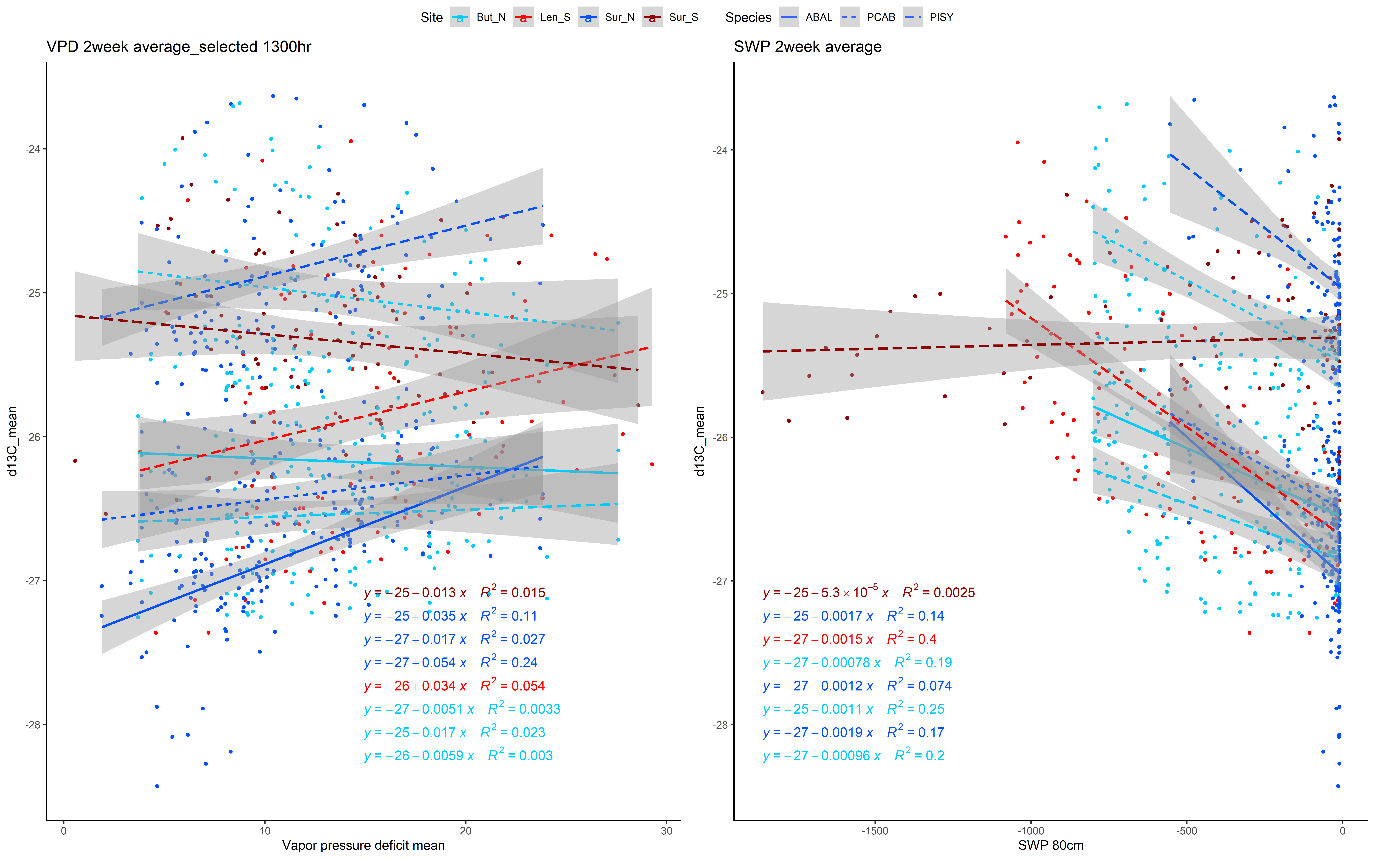


Fig.S.8 Non-aligned site and species mean δ^13^C shot values and their relationship with 2 week averages of vapour pressure deficit (VPD) for April to September.


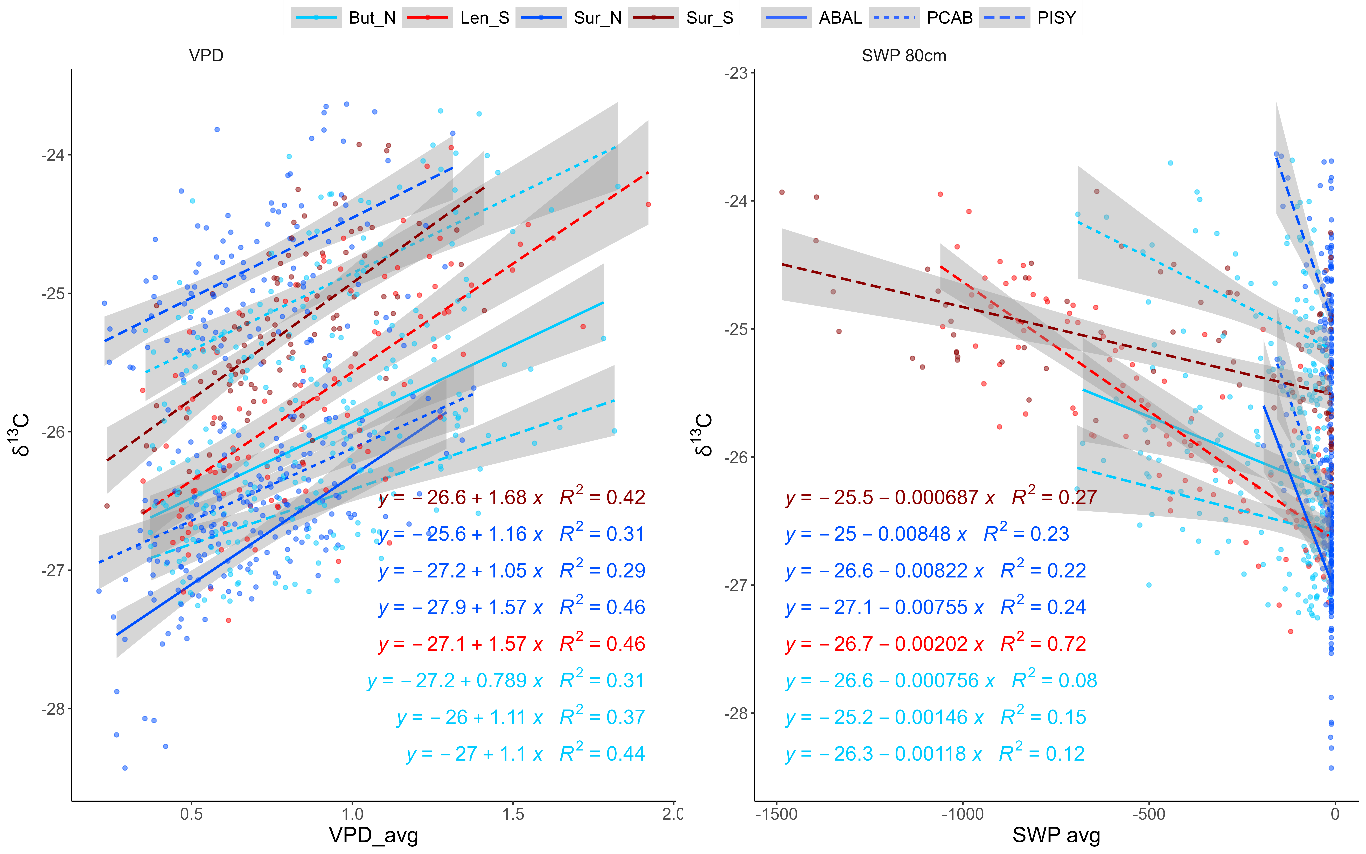


Fig.S.9 Site and species mean δ^13^C shot values and their relationship with the sequence-dependent averages of vapour pressure deficit (VPD) and soil water potential (SWP) at 80 cm depth, according to the selected window sequence and aligned to Day0 and End date. Linear regressions were fitted for all species and sites, and shot number is indicated by the points’ shape. Model equations and explained variance (R^2^) are indicated and follow the species order for each site.


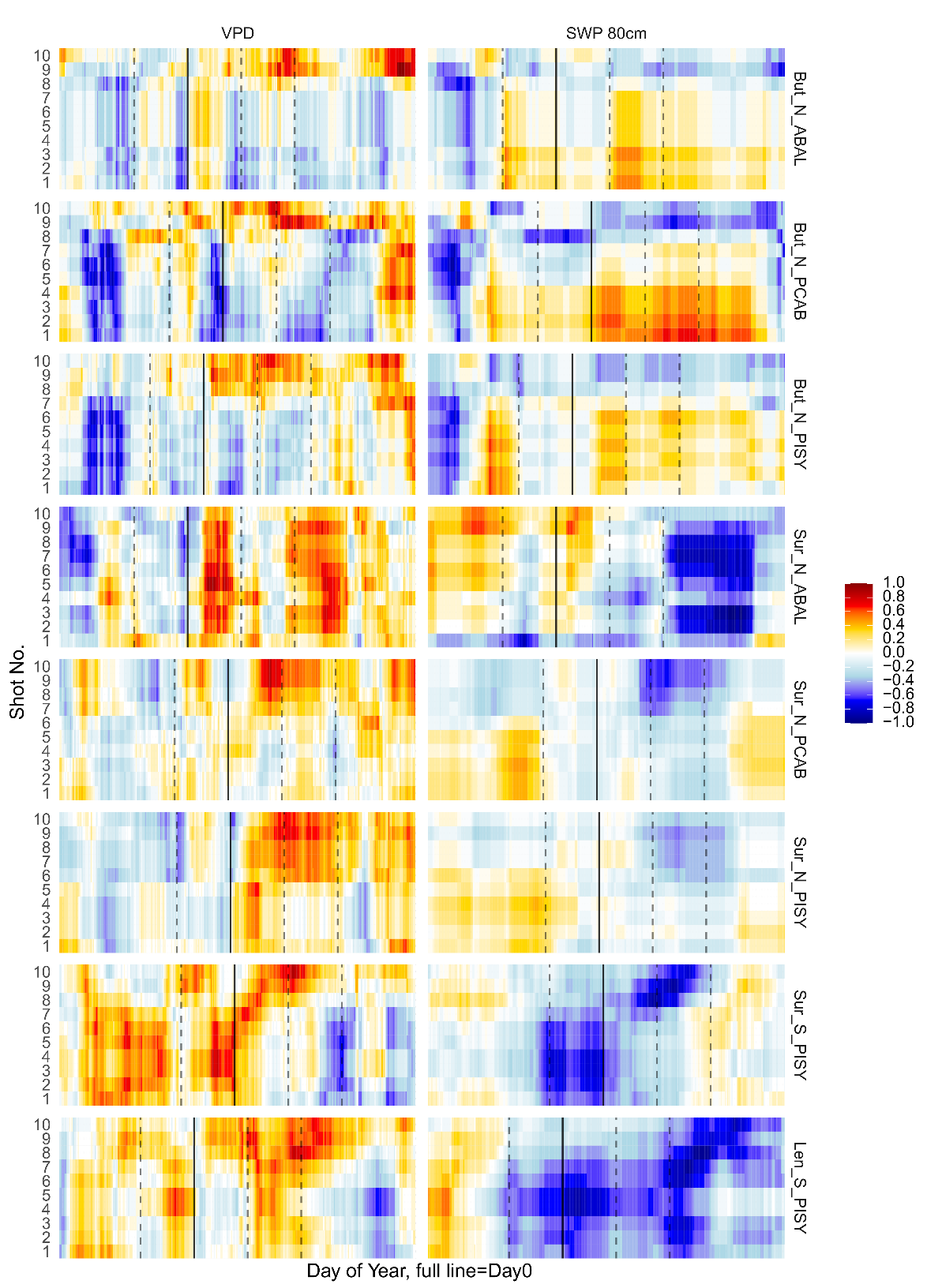
Fig.S. 10 Heatmaps displaying Kendall’s tau correlation coefficients between δ^13^C values measured in the 10 consecutive shots in tree-rings (y-axes) and vapour pressure deficit (VPD, left panels) and soil water potential at 80 cm depth (SWP 80 cm, right panels), aggregated into 20-day moving windows. The x-axis represents the day of the year, with solid vertical lines indicating Day 0 and dashed lines indicating 50 days before and 50 and 100 days after Day0. The staggering of the solid line across the panels reflects the difference in the start date for growth among the species at each site.


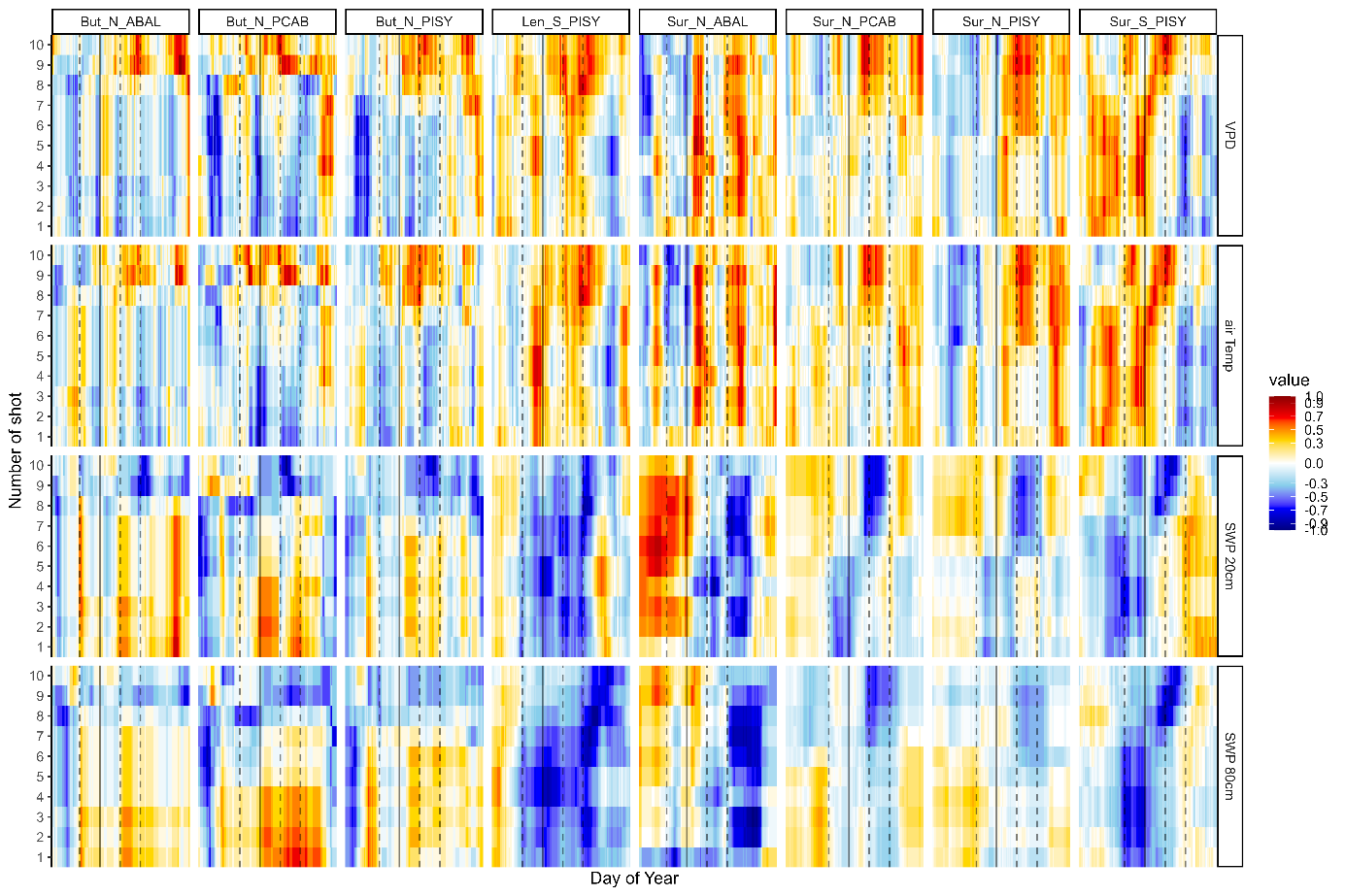


Fig.S.11 Heatmaps displaying the Kendall’s tau correlation coefficients between δ^13^C values, measured in the 10 consecutive shots in tree rings, and environmental variables, by 20-day aggregated windows. The x-axis shows the day of the year, with solid vertical lines indicating Day0 and dashed lines indicating 50 days before and 50 and 100 days after Day0. The staggering of the solid line across the panels reflects the difference in the start date for growth among the species at each site.


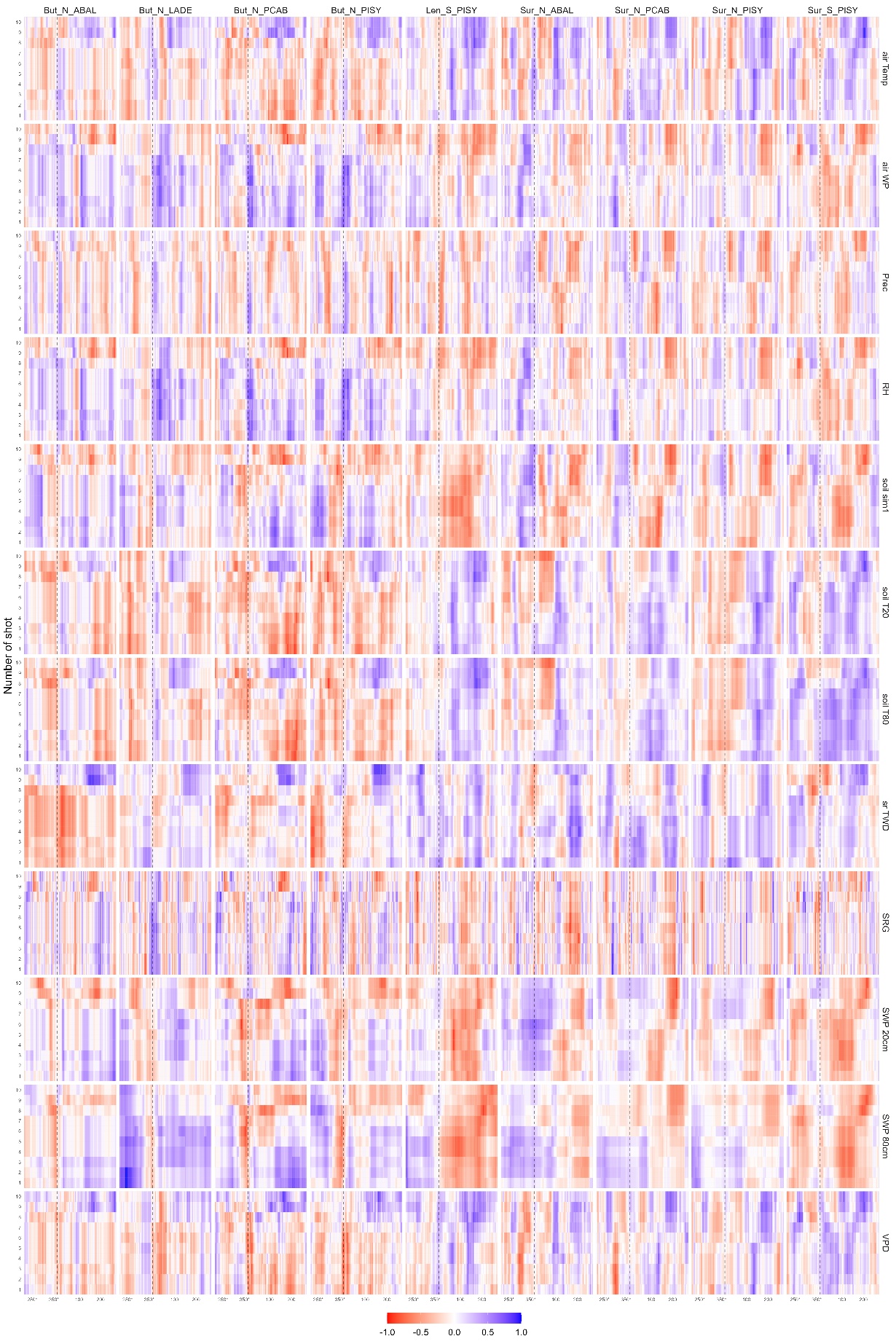


Fig.S.12 Heatmaps displaying the Kendall’s tau correlation coefficients between δ^13^C values measured in the 10 consecutive shots in tree rings, and environmental variables (Air Temperature= Airtemp, Air Water Potential=Airwp, Precipitation Summ= Prec, Relative Humidity= RH, Simulated Soil Moisture At 1cm= Soilsim1, Soil Temperature At 20cm Depth= Soil T20cm, Soil Temperature At 80cm Depth= Soil T80cm, Stem Growth = SRG, Soil Water Potential At 20 Cm Depth= SWP20cm, Soil Water Potential At 80cm Depth= SWP80cm, Vapour Pressure Deficit = VPD) by 20-day aggregated windows. The x-axis represents the calendar day of the start of the moving window.


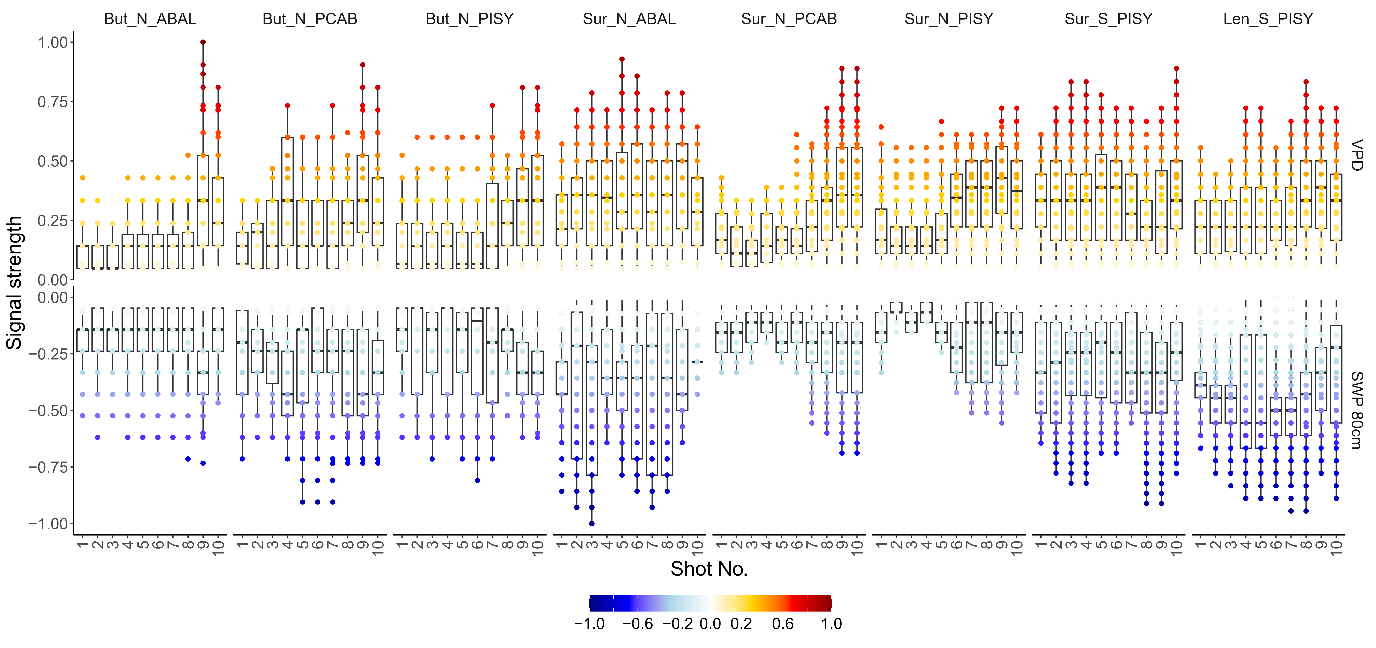
Fig.S.13 Kendall’s tau correlation coefficients (extracted from Fig.5) between δ^13^C values of the 10 consecutive shots for the measured years in tree rings for each site and species. We selected positive correlations for vapour pressure deficit (VPD, top panels) and negative correlations for soil water potential at 80 cm depth (SWP 80cm, bottom panels). Analysis of variance and post-hoc test results are given in Table S.4. Differences are visible between the first and last part of the ring, and significant changes at the 4^th^ and 8^th^ shots.


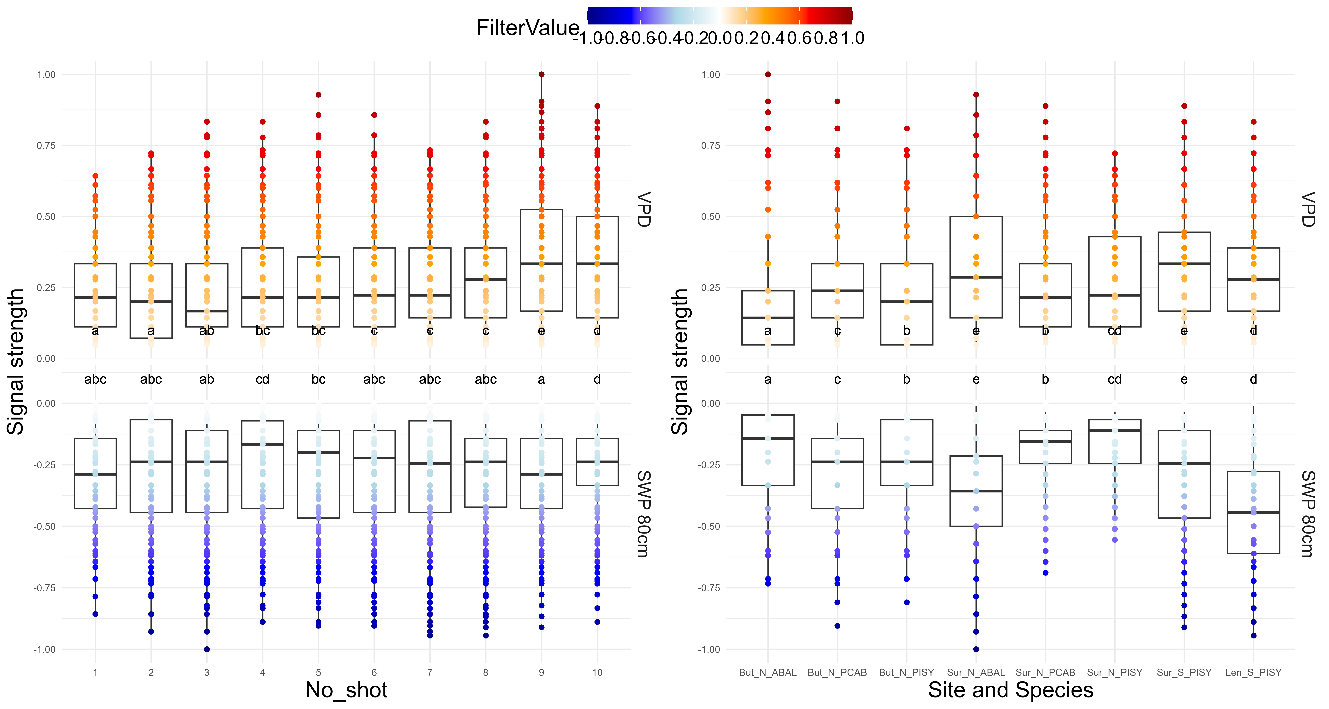


Fig.S.14 Differences (a) between the shots (across all sites and species, with particoular changes in significnace groups visible between the first and last part of the ring, with changes at the 4^th^ and 8^th^ shots.) and (b) between the site and species in the correlations between δ^13^C and environmental variables, restricted to positive correlations for vapour pressure deficit (VPD) and negative correlations for soil water potential (SWP) at 80 cm depth. Different lowercase letters indicate significant differences (ANOVA and post-hoc tests, P<0.05).

Table S. 5 Tukey’s honestly significant difference (HSD) test results for Fig. 6, showing Kendall’s tau correlation coefficients between δ^13^C shots for each site and species, for vapour pressure deficit (VPD) and soil water potential at 80 cm depth (SWP). Estimated marginal means (emmean) are given, with different lowercase letters indicating statistically significant differences (P<0.05).

| But_N_ABAL | | |  | | |  | | |  | | | Sur_N_PCAB | | |  | | |  | | | | |
| --- | --- | --- | --- | --- | --- | --- | --- | --- | --- | --- | --- | --- | --- | --- | --- | --- | --- | --- | --- | --- | --- | --- |
| No_shot | variable | | | emmean | | | Letters | | |  | | | No_shot | | | variable | | | emmean | | Letters |  |
|  |  | | |  | | |  | | |  | | |  | | |  | | |  | |  |  |
| 1 | VPD | | | 0.^13^8 | | | a | | |  | | | 1 | | | VPD | | | 0.191 | | ab |  |
| 2 | VPD | | | 0.091 | | | a | | |  | | | 2 | | | VPD | | | 0.^13^9 | | a |  |
| 3 | VPD | | | 0.1 | | | a | | |  | | | 3 | | | VPD | | | 0.^13^ | | a |  |
| 4 | VPD | | | 0.147 | | | a | | |  | | | 4 | | | VPD | | | 0.168 | | a |  |
| 5 | VPD | | | 0.147 | | | a | | |  | | | 5 | | | VPD | | | 0.17 | | a |  |
| 6 | VPD | | | 0.147 | | | a | | |  | | | 6 | | | VPD | | | 0.17 | | a |  |
| 7 | VPD | | | 0.147 | | | a | | |  | | | 7 | | | VPD | | | 0.233 | | b |  |
| 8 | VPD | | | 0.^13^9 | | | a | | |  | | | 8 | | | VPD | | | 0.3 | | c |  |
| 9 | VPD | | | 0.368 | | | c | | |  | | | 9 | | | VPD | | | 0.389 | | d |  |
| 10 | VPD | | | 0.294 | | | b | | |  | | | 10 | | | VPD | | | 0.389 | | d |  |
| 1 | SWP | | | -0.193 | | | b | | |  | | | 1 | | | SWP | | | -0.169 | | cd |  |
| 2 | SWP | | | -0.182 | | | b | | |  | | | 2 | | | SWP | | | -0.175 | | bcd |  |
| 3 | SWP | | | -0.203 | | | b | | |  | | | 3 | | | SWP | | | -0.^13^3 | | d |  |
| 4 | SWP | | | -0.166 | | | b | | |  | | | 4 | | | SWP | | | -0.114 | | d |  |
| 5 | SWP | | | -0.166 | | | b | | |  | | | 5 | | | SWP | | | -0.162 | | cd |  |
| 6 | SWP | | | -0.166 | | | b | | |  | | | 6 | | | SWP | | | -0.^13^8 | | d |  |
| 7 | SWP | | | -0.166 | | | b | | |  | | | 7 | | | SWP | | | -0.208 | | bc |  |
| 8 | SWP | | | -0.211 | | | b | | |  | | | 8 | | | SWP | | | -0.228 | | ab |  |
| 9 | SWP | | | -0.311 | | | a | | |  | | | 9 | | | SWP | | | -0.26 | | a |  |
| 10 | SWP | | | -0.181 | | | b | | |  | | | 10 | | | SWP | | | -0.26 | | a |  |
|  |  | | |  | | |  | | |  | | |  | | |  | | |  | |  |  |
| But_N_PCAB | |  | | |  | | |  | | | Sur_N_PISY | | |  | | |  | | |  | | |
| No_shot | variable | | | emmean | | | Letters | | |  | | | No_shot | | | variable | | | emmean | | Letters |  |
|  |  | | |  | | |  | | |  | | |  | | |  | | |  | |  |  |
| 1 | VPD | | | 0.167 | | | a | | |  | | | 1 | | | VPD | | | 0.218 | | a |  |
| 2 | VPD | | | 0.165 | | | a | | |  | | | 2 | | | VPD | | | 0.174 | | a |  |
| 3 | VPD | | | 0.209 | | | ab | | |  | | | 3 | | | VPD | | | 0.174 | | a |  |
| 4 | VPD | | | 0.33 | | | de | | |  | | | 4 | | | VPD | | | 0.174 | | a |  |
| 5 | VPD | | | 0.21 | | | ab | | |  | | | 5 | | | VPD | | | 0.207 | | a |  |
| 6 | VPD | | | 0.233 | | | abc | | |  | | | 6 | | | VPD | | | 0.334 | | b |  |
| 7 | VPD | | | 0.222 | | | ab | | |  | | | 7 | | | VPD | | | 0.357 | | b |  |
| 8 | VPD | | | 0.254 | | | bc | | |  | | | 8 | | | VPD | | | 0.357 | | b |  |
| 9 | VPD | | | 0.366 | | | e | | |  | | | 9 | | | VPD | | | 0.418 | | c |  |
| 10 | VPD | | | 0.286 | | | cd | | |  | | | 10 | | | VPD | | | 0.364 | | bc |  |
| 1 | SWP | | | -0.256 | | | cd | | |  | | | 1 | | | SWP | | | -0.^13^9 | | cd |  |
| 2 | SWP | | | -0.249 | | | cd | | |  | | | 2 | | | SWP | | | -0.056 | | e |  |
| 3 | SWP | | | -0.283 | | | abcd | | |  | | | 3 | | | SWP | | | -0.095 | | de |  |
| 4 | SWP | | | -0.376 | | | ab | | |  | | | 4 | | | SWP | | | -0.059 | | e |  |
| 5 | SWP | | | -0.274 | | | cd | | |  | | | 5 | | | SWP | | | -0.143 | | cd |  |
| 6 | SWP | | | -0.253 | | | d | | |  | | | 6 | | | SWP | | | -0.2^13^ | | ab |  |
| 7 | SWP | | | -0.281 | | | cd | | |  | | | 7 | | | SWP | | | -0.167 | | abc |  |
| 8 | SWP | | | -0.321 | | | abc | | |  | | | 8 | | | SWP | | | -0.167 | | abc |  |
| 9 | SWP | | | -0.36 | | | a | | |  | | | 9 | | | SWP | | | -0.208 | | a |  |
| 10 | SWP | | | -0.309 | | | bc | | |  | | | 10 | | | SWP | | | -0.161 | | bc |  |
|  |  | | |  | | |  | | |  | | |  | | |  | | |  | |  |  |
| But_N_PISY |  | | |  | | |  | | |  | | | Sur_S_PISY | | |  | | |  | |  |  |
| No_shot | variable | | | emmean | | | Letters | | |  | | | No_shot | | | variable | | | emmean | | Letters |  |
|  |  | | |  | | |  | | |  | | |  | | |  | | |  | |  |  |
| 1 | VPD | | | 0.143 | | | a | | |  | | | 1 | | | VPD | | | 0.32 | | bcd |  |
| 2 | VPD | | | 0.168 | | | ab | | |  | | | 2 | | | VPD | | | 0.341 | | bcd |  |
| 3 | VPD | | | 0.149 | | | a | | |  | | | 3 | | | VPD | | | 0.33 | | bcd |  |
| 4 | VPD | | | 0.168 | | | ab | | |  | | | 4 | | | VPD | | | 0.33 | | bcd |  |
| 5 | VPD | | | 0.149 | | | a | | |  | | | 5 | | | VPD | | | 0.373 | | d |  |
| 6 | VPD | | | 0.^13^7 | | | a | | |  | | | 6 | | | VPD | | | 0.347 | | bcd |  |
| 7 | VPD | | | 0.233 | | | bc | | |  | | | 7 | | | VPD | | | 0.302 | | abc |  |
| 8 | VPD | | | 0.245 | | | c | | |  | | | 8 | | | VPD | | | 0.256 | | a |  |
| 9 | VPD | | | 0.319 | | | d | | |  | | | 9 | | | VPD | | | 0.293 | | ab |  |
| 10 | VPD | | | 0.344 | | | d | | |  | | | 10 | | | VPD | | | 0.353 | | cd |  |
| 1 | SWP | | | -0.176 | | | d | | |  | | | 1 | | | SWP | | | -0.321 | | ab |  |
| 2 | SWP | | | -0.222 | | | bcd | | |  | | | 2 | | | SWP | | | -0.319 | | ab |  |
| 3 | SWP | | | -0.239 | | | bcd | | |  | | | 3 | | | SWP | | | -0.312 | | ab |  |
| 4 | SWP | | | -0.222 | | | bcd | | |  | | | 4 | | | SWP | | | -0.312 | | ab |  |
| 5 | SWP | | | -0.239 | | | bcd | | |  | | | 5 | | | SWP | | | -0.264 | | c |  |
| 6 | SWP | | | -0.234 | | | bcd | | |  | | | 6 | | | SWP | | | -0.279 | | bc |  |
| 7 | SWP | | | -0.246 | | | abc | | |  | | | 7 | | | SWP | | | -0.287 | | bc |  |
| 8 | SWP | | | -0.201 | | | cd | | |  | | | 8 | | | SWP | | | -0.362 | | a |  |
| 9 | SWP | | | -0.282 | | | ab | | |  | | | 9 | | | SWP | | | -0.356 | | a |  |
| 10 | SWP | | | -0.3 | | | a | | |  | | | 10 | | | SWP | | | -0.276 | | bc |  |
|  |  | | |  | | |  | | |  | | |  | | |  | | |  | |  |  |
| Sur_N_ABAL | |  | | |  | | |  | | | Len_S_PISY | | |  | | |  | | |  | | |
| No_shot | variable | | | emmean | | | Letters | | |  | | | No_shot | | | variable | | | emmean | | Letters |  |
|  |  | | |  | | |  | | |  | | |  | | |  | | |  | |  |  |
| 1 | VPD | | | 0.242 | | | a | | |  | | | 1 | | | VPD | | | 0.223 | | a |  |
| 2 | VPD | | | 0.335 | | | c | | |  | | | 2 | | | VPD | | | 0.231 | | a |  |
| 3 | VPD | | | 0.37 | | | cd | | |  | | | 3 | | | VPD | | | 0.228 | | a |  |
| 4 | VPD | | | 0.343 | | | c | | |  | | | 4 | | | VPD | | | 0.261 | | a |  |
| 5 | VPD | | | 0.386 | | | cd | | |  | | | 5 | | | VPD | | | 0.261 | | a |  |
| 6 | VPD | | | 0.388 | | | cd | | |  | | | 6 | | | VPD | | | 0.253 | | a |  |
| 7 | VPD | | | 0.327 | | | bc | | |  | | | 7 | | | VPD | | | 0.274 | | a |  |
| 8 | VPD | | | 0.334 | | | bc | | |  | | | 8 | | | VPD | | | 0.355 | | b |  |
| 9 | VPD | | | 0.422 | | | d | | |  | | | 9 | | | VPD | | | 0.36 | | b |  |
| 10 | VPD | | | 0.271 | | | ab | | |  | | | 10 | | | VPD | | | 0.331 | | b |  |
| 1 | SWP | | | -0.406 | | | abc | | |  | | | 1 | | | SWP | | | -0.394 | | c |  |
| 2 | SWP | | | -0.321 | | | de | | |  | | | 2 | | | SWP | | | -0.451 | | b |  |
| 3 | SWP | | | -0.422 | | | ab | | |  | | | 3 | | | SWP | | | -0.463 | | ab |  |
| 4 | SWP | | | -0.3 | | | e | | |  | | | 4 | | | SWP | | | -0.462 | | ab |  |
| 5 | SWP | | | -0.396 | | | abc | | |  | | | 5 | | | SWP | | | -0.462 | | ab |  |
| 6 | SWP | | | -0.44 | | | a | | |  | | | 6 | | | SWP | | | -0.508 | | a |  |
| 7 | SWP | | | -0.377 | | | bc | | |  | | | 7 | | | SWP | | | -0.508 | | a |  |
| 8 | SWP | | | -0.411 | | | abc | | |  | | | 8 | | | SWP | | | -0.467 | | ab |  |
| 9 | SWP | | | -0.363 | | | cd | | |  | | | 9 | | | SWP | | | -0.374 | | cd |  |
| 10 | SWP | | | -0.275 | | | e | | |  | | | 10 | | | SWP | | | -0.328 | | d |  |
